# Disease dynamics in a stochastic network game: a little empathy goes a long way in averting outbreaks

**DOI:** 10.1038/srep44122

**Published:** 2017-03-14

**Authors:** Ceyhun Eksin, Jeff S. Shamma, Joshua S. Weitz

**Affiliations:** 1 School of Biological Sciences, Georgia Institute of Technology, Atlanta, GA, USA; 2 School of Electrical and Computer Engineering, Georgia Institute of Technology, Atlanta, GA, USA; 3 Computer, Electrical and Mathematical Sciences and Engineering, King Abdullah University of Science and Technology (KAUST), Thuwal, Saudi Arabia; 4 School of Physics, Georgia Institute of Technology, Atlanta, GA, USA.

## Abstract

Individuals change their behavior during an epidemic in response to whether they and/or those they interact with are healthy or sick. Healthy individuals may utilize protective measures to avoid contracting a disease. Sick individuals may utilize preemptive measures to avoid spreading a disease. Yet, in practice both protective and preemptive changes in behavior come with costs. This paper proposes a stochastic network disease game model that captures the self-interests of individuals during the spread of a susceptible-infected-susceptible disease. In this model, individuals strategically modify their behavior based on current disease conditions. These reactions influence disease spread. We show that there is a critical level of concern, i.e., empathy, by the sick individuals above which disease is eradicated rapidly. Furthermore, we find that risk averse behavior by the healthy individuals cannot eradicate the disease without the preemptive measures of the sick individuals. Empathy is more effective than risk-aversion because when infectious individuals change behavior, they reduce all of their potential infections, whereas when healthy individuals change behavior, they reduce only a small portion of potential infections. This imbalance in the role played by the response of the infected versus the susceptible individuals on disease eradication affords critical policy insights.

Infectious diseases change social interaction patterns. During the Ebola outbreak, many studies pointed to changes in social customs playing a critical role in impeding disease spread, e.g., a switch to safe burial methods from traditional ceremonial burials^1^. Similar behavioral responses played important roles in modifying disease spread in other pandemics, e.g., wearing protective masks during the SARS pandemic^2^,^3^,^4^, decrease in unprotected sex when STD is at high levels^5^,^6^ or covering one’s own cough and staying at home if sick during a flu pandemic^7^,^8^,^9^. These responses to disease prevalence can, in turn, preempt disease spread by the infected to the susceptible individuals in the population.

In many infectious diseases infected and susceptible individuals have to be in close proximity for disease transmission. Accordingly, there has been a surge in interest on disease spread models in which a contact network determines the subset of individuals that can be infected by an infected individual^10^,^11^,^12^,^13^,^14^,^15^. These studies continue to be influential in relating network structural properties to outbreak thresholds and in revealing the limits to inferences made by models that assume homogeneous mixing.

Beyond network structure, the rate at which individuals meet with their contacts depends on the individual preemptive measures taken during the course of a disease^16^. Consequently, a number of dynamic models have been developed to assess the effects individual preemptive measures have on infectious disease spread over networks^17^,^18^,^19^,^20^. These models couple behavior and disease dynamics. That is, the state of the disease and the contact network determine the preemptive measures of the individuals which then affect the disease spread. Preemptive measures in these models, which are in the form of social distancing or rewiring of transmissive links, are assumed to be results of simple heuristics that approximate the decision-making of healthy individuals. These heuristic based decision-making algorithms are intended to be approximations of decisions made by self-interested individuals.

When individuals act according to their own self-interests, they compare the inherent costs of preemptive measures with the risks of disease contraction. However, the actions of other individuals also affect the risk of infection. Game theory provides a means to consider how individuals make rational decisions by reasoning strategically about the decisions of others. Recent epidemiological models with game theoretic individual decision-making either consider one-shot rational decisions of all susceptible individuals at the beginning, e.g., vaccinate or not, social distance or not, that determines the course of the disease^21^,^22^,^23^,^24^,^25^,^26^, or use bounded rational heuristics for repeated decision-making^27^,^28^,^29^,^30^ (see ref. 31 for a recent extensive review).

Here, we consider individuals—susceptible and infected— making daily rational decisions on preemptive measures, e.g., social distancing, staying home from school/work, wearing protective masks. These preemptive measures are based on the current risks of disease spread within a susceptible-infected-susceptible (SIS) model. In particular, a healthy individual compares the cost of protection measures with the current risk of infection. This means a healthy individual can forgo any protective measure (free-ride) if the individual perceives their sick contacts are taking the utmost preemptive measures. However, at the same time, a sick individual compares the cost of preemptive measures with the current risk of spreading the disease to their healthy neighbors. This means sick individuals have to reason strategically about the decisions of their healthy neighbors who reason about the decisions of their sick neighbors. This sets up a daily game among healthy and sick individuals. The daily rational measures taken by both the healthy and the sick as a result of the *disease network game* set the probabilities of disease contractions which in turn stochastically determine the status of the disease in the following day.

Using this model, we explore the interrelationship among contact network structure, individual behavior, and disease spread dynamics. Our goal is to determine how behavior can change whether a disease will become endemic or not. In doing so, we explore the significant, and differential effects of empathy vs. risk aversion.

## Results

### Disease spread over a contact network

**Figure 1:**
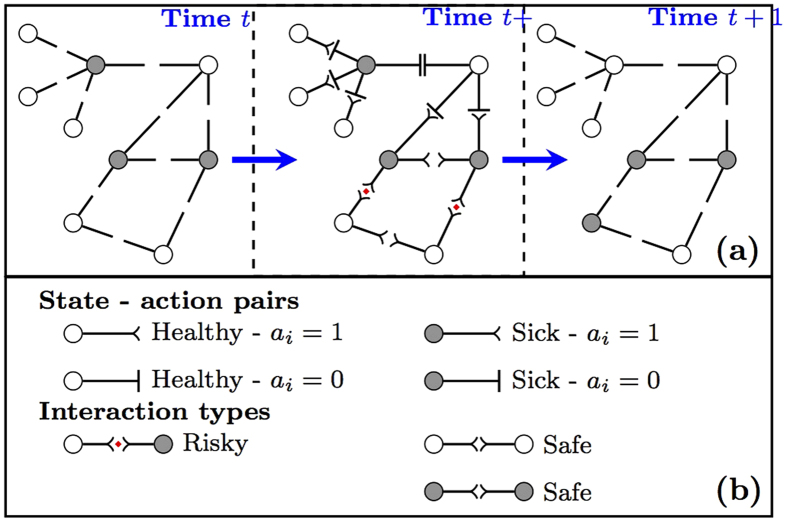
SIS Markov chain dynamics. We show one step of the Markov chain dynamics in (**a**) where circles denote individuals who are healthy (open) or sick (shaded). The edges denote contacts, where the actions are indicated by the edge-end types, social distancing | and interaction 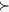. Given the state of the disease and contact network at time t, individuals decide to take preemptive measures or not at time *t*+ which determines the state of each individual at time *t* + 1 according to the Markov chain dynamics in (7). If *a*_*i*_ = 1, individual *i* does not take any preemptive measures. If *a*_*i*_ = 0, *i* self-isolates reducing any risk of disease contraction or spread to zero. A healthy individual can only contract the disease from an interaction with a neighbor if and only if the individual’s neighbor is infected and neither of the two self-isolates. These interactions are marked by a red dot in the middle figure. We enumerate each pair of state and action in (**b**).

We develop an individual based game-theoretic model that captures the interests of healthy and sick individuals during an epidemic. Full details appear in the Methods section. At any point in time, the population consists of sick and healthy individuals. Healthy individuals are susceptible to contract the disease in the next time step if in contact with an infected individual. Disease contraction from a sick contact depends on the inherent infectivity of the disease denoted by *β*. Inherent infectivity level of a disease is reduced by the combined measures taken by the two individuals in contact. Protective measures taken by a healthy individual *i*, denoted by *a*_*i*_, ranges from 0 –self-isolate– to 1 –resuming normal activity. Similarly, preemptive measures taken by a sick individual *j*, denoted by *a*_*j*_, ranges from 0 –self-isolate– to 1 –resuming normal activity. Individual *i* contracts the disease from a sick individual *j* with probability *βa*_*i*_*a*_*j*_. That is, infection can only occur if neither the healthy nor the sick individual decides to self-isolate, i.e., *a*_*i*_ > 0 and *a*_*j*_ > 0. The set of contacts of each individual defines a contact network. Accordingly, we refer to the set of contacts of an individual as their neighbors. A healthy individual remains healthy in the next time step if the individual does not contract the disease from any of their sick neighbors. That is, disease transmission from one sick neighbor is enough to infect the individual. A sick individual heals with probability *δ* and becomes susceptible again. See Fig. 1 for an example of a contact network and one step propagation of the SIS Markov chain model.

Individuals determine their actions at each time with respect to whether they are healthy or sick, and the states (healthy or sick) and actions of their neighbors. On the one hand, a healthy individual would like to avoid taking costly protective measures while minimizing their risk of infection. On the other hand, a sick individual would like to avoid taking costly preemptive measures while minimizing the chance of transmitting the disease to their healthy neighbors. We represent the tradeoffs of an individual by a payoff function which is a weighted combination of these preferences (see Equation (9) in the Methods). We represent the cost of measures by a socialization term that an individual maximizes when resuming normal activity. We weight this socialization term by a positive *socialization* constant *c*_0_. The risk of infection of a healthy individual, weighted by a positive *risk averseness* constant *c*_1_, increases with the number of sick neighbors that do not take any preemptive measures. The concern for disease transmission, weighted by a positive *empathy* constant *c*_2_, increases with the number of healthy neighbors who do not take any protective measures.

Given the payoffs described above with fixed weight constants, *c*_0_, *c*_1_, and *c*_2_, individuals make decisions based on observations of the current state of the disease. In addition to the state of contact neighbors, a healthy individual’s risk of infection or a sick individual’s chance of disease transmission, and therefore, individual’s payoff, depends on the actions of contact neighbors. For instance, a healthy individual is safe from the disease regardless of their own action if all of their sick neighbors self-isolate. That is, a healthy individual can avoid the disutility of protective measures by resuming normal activity if the individual knows their infected neighbors are taking preemptive measures to avoid disease spread. However, their sick neighbors also would like to avoid the disutility of these preemptive measures. Hence, this is a game amongst healthy and sick individuals of the population. In this game, at each stage individuals have to reason about the actions of their neighbors to make their own decisions.

### Deriving optimal strategic behavior in a disease network game

The disease dynamics described above and depicted in Fig. 1 are a stochastic game^32^. Given a state of the disease at time *t* individuals in the population make decisions regarding their payoffs, which then determine the disease contraction probabilities *βa*_*i*_*a*_*j*_ of healthy individuals. These transition probabilities along with the state of the disease at time *t* determine the state of the disease at time *t* + 1. In general, stochastic games denotes a class of games where the current state determines the payoffs of players, and the actions taken in the current time step affect the probabilistic transition to the state in the following time.

A rational model of decision-making in a stochastic game is the Markov perfect equilibrium solution concept [ref. 33, Ch.5.5]. A Markov strategy is where individuals’ actions depend only on the payoff relevant state of the disease. In addition, we assume individuals take actions considering their current payoffs only. This equilibrium concept, we term the myopic Markov perfect equilibrium (MMPE), is formally defined in the Methods section. An MMPE strategy profile at time *t* is such that no individual has a preferable unilateral deviation to another action that strictly increases the individual’s current payoff. The assumption of myopic strategies implies that individuals do not weigh their future risks of infection or infecting other individuals in their current decision-making. This is a reasonable assumption considering the computational complexity of accounting for future states of the disease during an epidemic.

**Figure 2:**
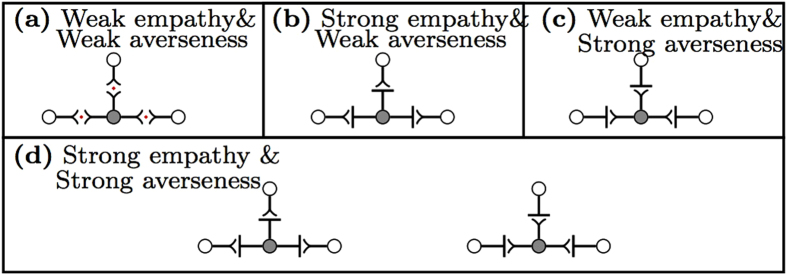
MMPE equilibrium strategy actions with respect to utility constants. There are *n* = 4 individuals forming a star network. In cases (**a**–**c**) the stage equilibrium action is unique. In case (**d**) there two stage equilibria. When risk averseness is weak, i.e., *c*_0_ > *c*_1_, all healthy individuals take action *a*_*i*_ = 1 regardless of the action of the center individual. When empathy is weak, i.e., *c*_0_ > 3*c*_2_, the sick individual at the center takes action *a*_*i*_ = 1 regardless of *i*’s neighbors’ actions by the same reasoning. Based on these responses we can solve for stage equilibrium in (**a**–**c**). In (**a**) (*c*_0_ > *c*_1_ and *c*_0_ > 3*c*_2_), all individuals take action 1. In (**b**), because all healthy individuals take action 1 due to weak averseness, the sick individual takes action 0 considering the strong empathy (*c*_0_ < 3*c*_2_). In (**c**), because the sick individual takes action 1 due to weak empathy, all healthy individuals take action 0 considering their strong averseness (*c*_0_ < *c*_1_). In (**d**), if healthy individuals take action 1 then it is in the interest of the strongly empathetic sick individual to take action 0. However, if healthy individuals take action 0 then the sick individual receives a positive payoff from taking action 1. In both cases no individual has an incentive to deviate to another action.

We exemplify different equilibrium actions that arise from differing payoff constants (*c*_0_, *c*_1_ and *c*_2_) on a *n* = 4 individual star network given the state of the disease, where the center individual is sick and other individuals are healthy in Fig. 2(a–d). We observe that in cases (a–c) the equilibrium action is unique. When individuals have strong risk aversion and strong empathy in comparison to the socialization constant in (d), there are two alternative stage equilibria: 1) All healthy individuals resume normal activity and the sick individual self-isolates, and 2) the sick individual resumes normal activity and healthy individuals self-isolate. Both equilibrium strategies yield the same outcome of disease eradication–each individual continues to take the same action until the center individual heals. However, the first equilibrium action profile yields an aggregate utility—sum of individual payoffs—of 3 while the second equilibrium action yields an aggregate utility of 1 yielding a ratio of 1/3. The ratio of the worst aggregate utility value attained by an equilibrium action profile to the optimal action profile that maximizes the aggregate utility is referred to as the price of anarchy in game theory^34^. In Supplementary Section B, we prove a lower bound for the price of anarchy that shows it can scale with 1/*n*. The set of utility constants in (d) shows that the bound is tight because the price of anarchy equals 1/(*n* − 1) = 1/3.

**Figure 3:**
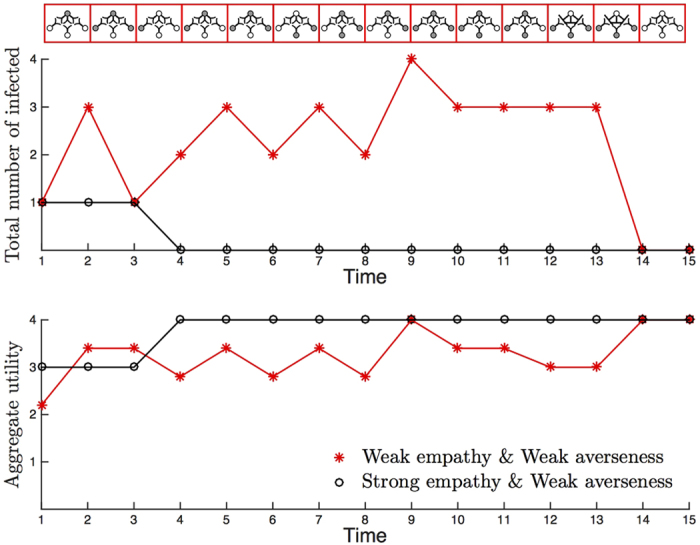
Behavior and disease dynamics with respect to payoff constants. The red and black lines in the figures correspond to payoff constants with weak and strong empathy, respectively. In both setups we have *β* = 0.4, *δ* = 0.2, *c*_0_ = 1 and weak averseness (*c*_0_ > *c*_1_). In particular, we consider risk aversion constant *c*_1_ to be 3*c*_1_ > *c*_0_ > 2*c*_1_. The MMPE action for the strong empathy case is given by Fig. 2(b). The MMPE action at time *t* = 1 for weak empathy is given by Fig. 2(a). The sequence of networks at the top shows the disease state and MMPE action of each individual at each time on the network for the weak empathy & weak averseness case. In this case, the MMPE actions are such that all individuals socialize at all times unless a healthy individual has three sick neighbors when the healthy individual self-isolates–see times 12 and 13. This is because of the value of the risk aversion constant obeys the relation 3*c*_1_ > *c*_0_. Bottom figure represents the corresponding aggregate utility for each case.

A disease is eradicated when all individuals are healthy. Starting at the state in Fig. 2 for utility constant values given by (b–d), there is no chance of disease spread at an MMPE strategy profile. Therefore, the disease is eradicated when the center individual heals in continuation of the disease dynamics. However, if constant values are as given by Fig. 2(a), there is no guarantee that the disease is eradicated when the center individual heals. We show the continuation of the disease dynamics starting from the cases of (a) weak empathy & weak averseness and (b) strong empathy & weak averseness in Fig. 3. In case (a), the disease takes off after the first time step in the network before eradication at time *t* = 14. When empathy is strong in (b), disease is eradicated when the center node heals. We also observe that the larger aggregate utility does not always lead to a reduction in disease spread. For instance at time *t* = 2 in Fig. 3 (bottom), the aggregate utility is higher in the weak empathy case than in the strong empathy case. At the following time *t* = 3 the number of infected increases to 3 individuals for the weak empathy case. At time *t* = 3, the aggregate payoff remains higher than the strong empathy case with single infected individual. That is, a larger epidemic size can lead to a better aggregate utility when the empathy constant is different.

In the example given in Fig. 3, the MMPE strategy profiles for different utility constants lead to qualitatively different outcomes. This means that rational behavior response can eradicate the disease rapidly depending on the sensitivity of individuals to risk of infection and concern for spreading the disease. In the following sections, we explore the effects of these constants on disease spread.

### Limits of disease spread from a single sick individual

The basic reproduction number *R*_0_ measures the spread of an infectious disease from an initial sick individual in an otherwise susceptible host population. In the homogeneously mixed SIS model, the basic reproduction number is equal to *R*_0_ = *βn/δ* [ref. 35, Ch. 2]. In this model, the disease becomes endemic if *R*_0_ > 1. In contact network disease models, *R*_0_ > 1 is not necessarily the outbreak threshold. Rather, it is an indicator that the disease is likely to persist when there are relatively low number of infected individuals^13^. Here, we compute an upper bound of the *R*_0_ value for the disease network game to relate the likelihood of disease persistence to network and utility constants.

Consider a network with degree distribution *P(k*) where *P(k*) denotes the probability that a selected individual has 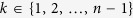 neighbors. We assume the initial infected individual is chosen from the population uniformly at random. When individuals act according to an MMPE strategy profile, we have the following bound on *R*_0_ (Supplementary Section C),


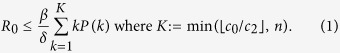


When the empathy term *nc*_2_ is smaller than *c*_0_, i.e., *K* = *n*, we recover the bound for contact network models without behavior response. The reasoning is as follows. An initial infected individual will choose to resume normal activity (*a*_*i*_ = 1) even given *n* − 1 susceptible neighbors, and no matter how these neighbors act because of weak empathy. When the empathy term is larger than the socialization constant, i.e., *c*_2_ > *c*_0_, the initial infected individual will self-isolate (*a*_*i*_ = 0) even given a single social neighbor. Therefore, the bound above is most interesting when the empathy term is such that *c*_0_/*n* < *c*_2_ < *c*_0_. In this case, only the infected individuals with connections smaller than 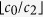 can spread the disease to their neighbors initially. The degree distribution *P*(·) determines the frequency of individuals with number of neighbors less than 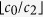. We note that all the susceptible individuals will socialize at normal levels initially if *c*_0_ > *c*_1_. Still, if the disease spreads to their neighbors, their rational actions can include self-isolation. In the derivation of the upper bound, we assume all susceptible neighbors of the initial infected individual resume normal activities. Hence the risk averseness constant *c*_1_ does not appear in the bound above. It is natural to think that high risk aversion may help reduce initial spread. Contrary to this intuition, when we explore the effects of *c*_1_ in the following sections, we find that the risk averseness cannot stop the initial spread without a critical level of the empathy constant.

We consider random scale-free networks to illustrate the conjoint effects of network structure and the empathy constant. In a scale-free network degree connectivity distribution follows power law, i.e., *P(k*) ~ *k*^−*γ*^, where 2 ≤ *γ* ≤ 3. When *γ* = 2, we obtain the following upper bound for *R*_0_ using (1) (Supplementary Section D),


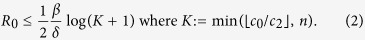


We observe how network and behavior response comes at play in the bound above. In particular, if the empathy constant is negligible, i.e., *c*_0_/*c*_2_ > *n*, then the bound increases logarithmically with the size of the population, i.e., *R*_0_ ≤ *β* log(*n*)/*δ*. This value is the reproduction number for the contact network SIS model with no individual behavior response to disease prevalence [ref. 36, Ch. 17]. We observe the effect of individual behavior on *R*_0_ as the empathy constant *c*_2_ is increased. The power law degree distribution of the scale-free network results in a logarithmic decrease in the bound with respect to increasing empathy constant *c*_2_. From (2) we have *R*_0_ < 1 for


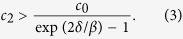


**Figure 4:**
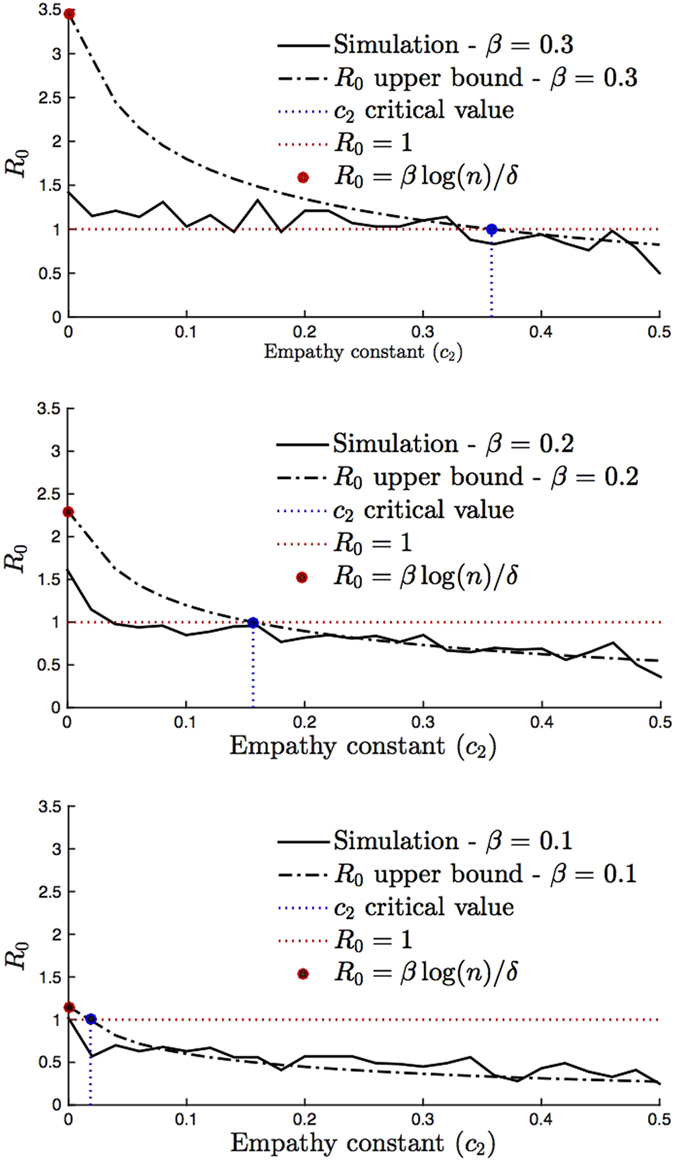
Accuracy of the critical empathy *c*_2_ threshold for *R*_0_ < 1. We consider *n* = 100 individuals and set the constants as *δ* = 0.2, *c*_0_ = 1 and *c*_1_ = 0.24. We let 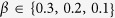 for top, middle, and bottom figures, respectively. The dotted dashed lines are the *R*_0_ upper bound value (2) with respect to the *c*_2_ value on *x*-axis. For *c*_2_ = 0, we have the red circled points corresponding to the *R*_0_ upper bound when there is no behavior response by the initial sick individual. Note that all the red circled points indicate *R*_0_ > 1. From (3), the critical values of *c*_2_ that make *R*_0_ < 1 equal to 0.02, 0.16, and 0.36 for 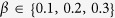, respectively. These points are marked in blue. *R*_0_ upper bound increases linear in *β* according to (2). We simulate *R*_0_ values as follows. We generate a scale-free network with *γ* = 2 according to the preferential attachment algorithm^38^. For each *β* and *c*_2_ value pair, we consider 100 realizations with randomly selecting patient zero and counting the number of individuals infected by patient zero until patient zero heals. Each point in the solid lines corresponds to the average of the total count values in 100 initializations. We observe that the simulated average *R*_0_ is less than one above the critical *c*_2_ value in (3).

We measure the accuracy of the critical *c*_2_ value above that makes *R*_0_ < 1 by comparing it to simulated *R*_0_ values in Fig. 4. Figure 4 shows the simulated *R*_0_ values and the upper bound in (2) with respect to the *c*_2_ value on the *x*-axis for a given *β* value. We see that for all *β* values when the upper bound is less than one, the *R*_0_ value obtained from simulation is also less than 1. That is, the disease is not likely to persist when the empathy constant of individuals is above the critical level in (3).

### Limits of disease spread from a secondary sick individual

Note that in the *R*_0_ definition above the sick individual is chosen randomly amongst all nodes. It is evident that *R*_0_ can be small in a scale-free network because many individuals have very few connections while a few individuals are highly connected. Highly connected individuals are less likely to be initially infected, however, it is likely that the initial infected individual is connected to a highly connected individual^13^. Hence, in the event that the initial individual infects a highly connected neighbor then the spread of the disease from a second infected individual can be fast. To account for this event, we consider the metric 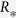 which is defined as the average number of new infections by an initial infected individual when patient zero is selected randomly weighted by their connectivity degree. Consider a network with degree connectivity distribution *P(k*). The probability that we select an infected individual with degree *k* is given by 
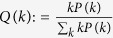
. This selection process represents the likely scenario that one of the earliest infected individuals transmits the disease to a highly connected individual. We have the following upper bound for the metric given individuals acting according to an MMPE action profile (Supplementary Section E)


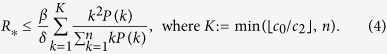


Note that the denominator of the term inside the sum is the average degree connectivity of the network. The numerator is the variance of the degree distribution when *K* = *n*. Comparing (1) with (4), it is possible that even though *R*_0_ < 1, it might be that 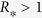 or vice versa. We elaborate on the differences between the two metrics further by considering the scale-free network where *P(k*) ~ *k*^−*γ*^ for *γ* = 2. We obtain the following upper bound for the 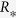 by the inequality above (Supplementary Section F),


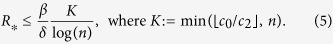


Comparing the bound above with the bound for *R*_0_ in (2), we observe the 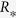 bound is more sensitive to the empathy constant. In particular, the decrease in the 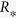 bound with respect to increasing *c*_2_ is linear while the decrease is logarithmic for the *R*_0_ bound. When *K* = *n*, the bound above grows with *n*/log(*n*) while the *R*_0_ bound grows with log(*n*). When 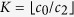, the 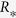 bound decreases with increasing *n* while the bound of *R*_0_ is not affected by the population size. From the bound above, we have 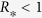 for


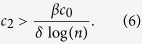


**Figure 5:**
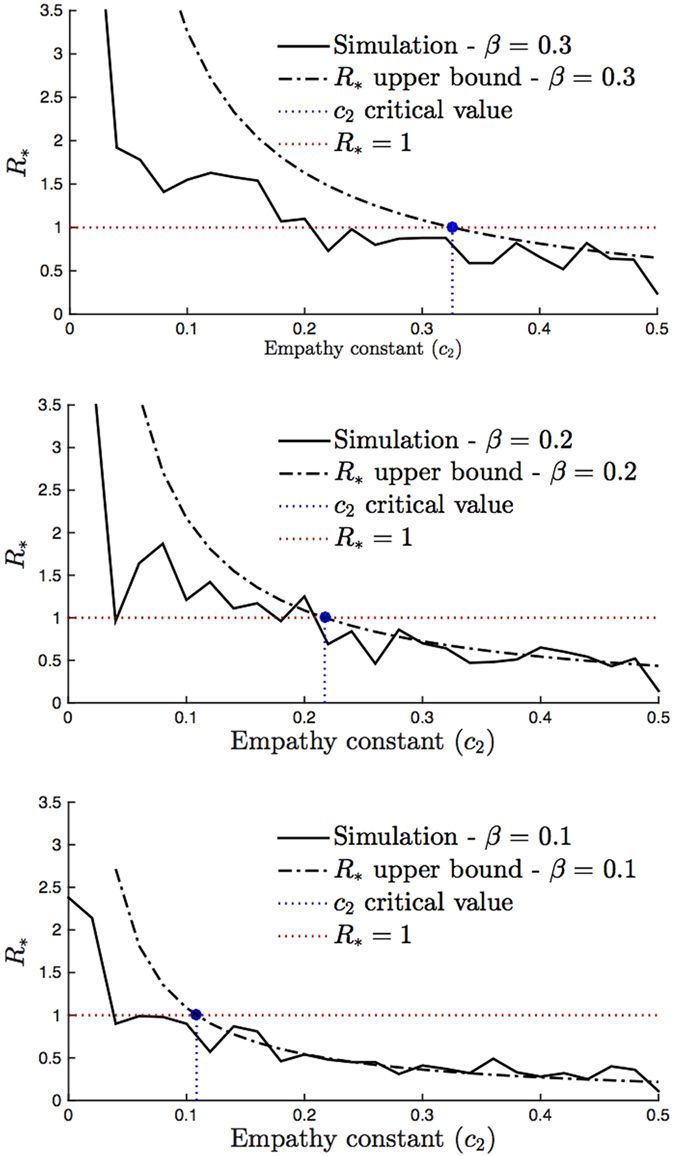
Accuracy of the critical empathy *c*_2_ threshold for 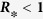. We consider *n* = 100 individuals and set the constants as *δ* = 0.2, *c*_0_ = 1 and *c*_1_ = 0.24. We let 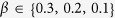 for top, middle, and bottom figures, respectively. The dotted dashed lines are the 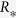 bound value in (5) with respect to the *c*_2_ value on the *x*-axis. The critical values of *c*_2_ in (6) that make 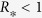 are 0.11, 0.22 and 0.33 respectively for 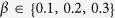. These points are marked in blue. We simulate 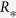 values identical to the way we simulate *R*_0_ values in Fig. 4 except that we select the initial sick individual according to distribution *Q(k*). We observe the simulated 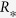 values are less than one for all *c*_2_ values above the critical value in (6). In comparison the critical *c*_2_ values for *R*_0_ in (3)–see Fig. 4–do not accurately predict the values of *c*_2_ above which 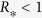.

We measure the accuracy of the critical *c*_2_ value above that makes 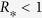 by comparing it to simulated 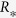 values in Fig. 5. Figure 5 shows the simulated 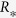 values where we select the initial infected individual with respect to *Q(k*), and the upper bound in (6) with respect to the *c*_2_ value on the *x*-axis for a given *β* value. We see that for all *β* values when the upper bound is less than one, i.e. when *c*_2_ is larger than the value in (6), the 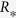 value obtained from simulation is also less than 1. That is, the disease is not likely to persist when the empathy constant of individuals is above the critical level in (6). In comparison, we observe that the *R*_0_ upper bound (2) is not an accurate upper bound for the simulated 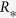 values. In particular, for small values of *β*, while the critical *c*_2_ value in (3) estimates disease is not likely to persist, it may persist according to the 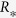 metric.

### *R*_0_ and 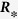 as endemic thresholds

*R*_0_ < 1 and 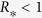 imply that the disease is likely to be eradicated before spreading to other individuals if initially there is a single sick individual. However, these conditions may not constitute a threshold for any initial state of the disease. The *R*_0_ value of the SIS model with homogeneous mixing gives us a general condition for disease eradication, *βn/δ* < 1. For SIS disease dynamics over networks when there is no individual response to disease prevalence, we obtain a disease eradication threshold of *βλ*_*max*_(*A*)/*δ* < 1 where *λ*_*max*_(*A*) is the largest eigenvalue of the adjacency matrix *A* of the contact network^14^.

Regarding these conditions, the more complex the model that the condition is derived from, the tighter the bound is. For instance, comparing the latter two bounds we have 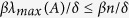. That is, the networked disease model makes a sharper prediction of disease eradication than the model with homogeneous mixing assumption. By the same reasoning, the thresholds based on the stochastic network disease game constitute sharper bounds for disease eradication when compared to the networked models with no behavior response. In particular, from the bounds for *R*_0_ and 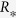 in the previous section, we know that there exist critical empathy constant values *c*_2_ in (3) and (6) which make these values less than one even when 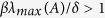.

**Figure 6:**
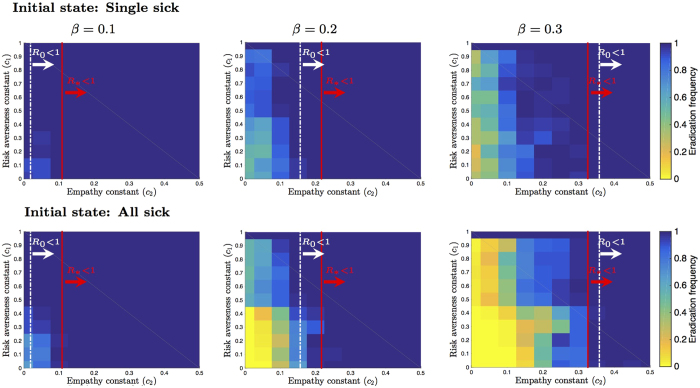
Effect of risk averseness *c*_1_ and empathy *c*_2_ constants on eradication. We consider *n* = 100 individuals, and let *δ* = 0.2, and *c*_0_ = 1. The infection rate *β* values equal to 0.1, 0.2 and 0.3 for figures left, middle, and right, respectively. The runs start with a single infected individual and all individuals infected respectively for the top and bottom figures. In each plot, the axes correspond to the constant values of *c*_1_ and *c*_2_. For a given value of *c*_1_ and *c*_2_, we generate 50 scale-free networks using the preferential attachment algorithm and run the stochastic network disease game for 200 steps for each network. The grid color represents the ratio of runs in which disease is eradicated within 200 steps. For figures left, middle, and right the eradication of threshold 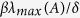 is equal to 2.65, 5.3, and 8, respectively. That is, 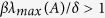 for all the figures. The critical values of the empathy constant, 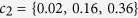 that make *R*_0_ < 1 for 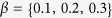 calculated using (6) are marked with white dotted dashed lines. The critical values of the empathy constant, 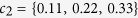, that make 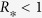 for 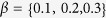 calculated using (6) are accurate in determining fast eradication for any value of *c*_1_ (marked with red solid lines).

In Fig. 6, we assess the accuracy of conditions for *R*_0_ < 1 and 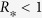 as indicators of disease eradication when compared to 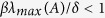. In the setup we consider disease parameter and network values where 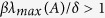, i.e., 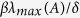 value equals 2.65, 5.3, and 8 for figures left, middle and right respectively. Furthermore, we consider two initial cases: 1) single infected (top) and 2) all infected (bottom). Figure 6 shows the frequency of runs that eradicated the disease before the simulation horizon for a given set of parameter values of *β, c*_1_ and *c*_2_. Our initial observation is that the outbreak threshold condition 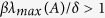 is not necessarily indicative of an endemic disease. That is, even though 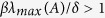, the disease can be eradicated depending on the empathy constant value *c*_2_. In addition we observe a direct relation between the frequency of disease eradication and the value of the empathy constant *c*_2_. The critical values of *c*_2_ that make 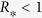 in (6) are indicative of disease eradication. We confirm these results for any value of risk averseness constant *c*_1_. That is, the critical values of *c*_2_ for which 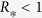 are indicators of disease eradication for all 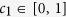. As *c*_2_ increases above the critical value, the average time to eradication decreases (see Supplementary Sections G and H for corresponding figures).

### The impact of risk averseness on disease spread

While the risk averseness constant *c*_1_ does not show up in any of our bounds for *R*_0_ and 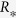, it can play a critical role in who takes preemptive measures as illustrated by the example in Fig. 2. Thus, the equilibrium infectivity level can depend on *c*_1_. Our first result regarding the risk averseness constant shows that when the empathy constant *c*_2_ is zero, we obtain the same outbreak threshold condition as when the behavior response is not accounted for in a disease spread model, i.e., 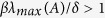 for any 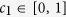. This threshold is analytically obtained by first approximating the Markov chain dynamics by a *n*-state differential equation and then linearizing the approximate model around its trivial fixed point, the origin – see Supplementary Section K. The derivation is similar to the derivation of the threshold for disease dynamics over networks without behavior response^14^. This result implies that no matter how risk averse the susceptible individuals are, they cannot eradicate the disease with certainty without the empathy of infected individuals in the stochastic disease network game.

Both this analytical result and the bounds for *R*_0_ and 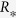 assume initially only a single individual is infected. Hence, these conditions might not be accurate when the infected individuals are large. In Fig. 6 (bottom) and in Supplementary Section J, we consider numerical simulations where the expected number of initial infected is {5%, 20%, 50%, 100%}. These numerical simulations confirm that the disease cannot be eradicated at any *c*_1_ value if 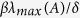 is large and the empathy constant is zero. For values of 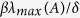 closer to 1, a high enough risk aversion helps to eliminate the disease Fig. 6 (left). Finally, we observe that the frequency of disease eradication increases as we increase the risk aversion constant *c*_1_ value when *c*_2_ is positive in Fig. 6.

This observation implies that even a little bit of empathy can go a long way in eradication of the disease given risk averse susceptible individuals. In other words, when the empathy term is a positive value *c*_2_ > 0, there exists a sufficiently high enough risk averseness constant that is likely to eradicate the disease. While it may be that the risk averseness by itself cannot eradicate the disease, we observe that it reduces the average number of infected individuals when the disease is endemic (see Supplementary Section H for corresponding figures). That is, risk averseness has longer-term effects on the disease dynamics comparable to empathy.

## Discussion

Behavior changes are ubiquitous during infectious disease outbreaks. Hence, accurate modeling of behavior has the potential to help with the prediction of disease’s impact and with the assessment of policy measures. To this end, we considered a stochastic network game where individuals respond to the current risk of disease spread, and their responses together with the current state of the disease and the contact network structure stochastically determine the next stage of the disease. In particular the game is played among the healthy and the sick in an SIS infectious disease. In our scenario, the concern for disease contraction of a healthy individual increased with the number of sick contacts that are not taking any preemptive measures. Similarly, sick individuals had increased concerns for disease spread when there are more healthy contacts that do not take protective measures. This meant that the incentives for a healthy individual taking a measure decreased as more of individual’s sick contacts took preemptive measures, e.g., staying at home. Similarly, the incentive for preemptive measures decreased for sick individuals as the healthy got more cautious. The consequences of these incentives are not trivial in a disease contact network setting where an individual cares about the behaviors of contacts who themselves care about their neighbors and so on. Hence, our analysis focused on the impact of rational behavior on disease spread.

Our results show that when individuals act rationally, there exists a level of concern by the infected individuals (empathy) above which the reproduction number (*R*_0_ and 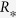) is less than one. In contrast, the risk aversion of healthy individuals is not a determinant of disease eradication when the empathy of the sick individuals is zero, i.e., when sick individuals are not responsive. Yet, for a positive level of empathy, there exists a risk averseness constant above which disease is likely to be eradicated. The intuition for these results is based two key observations. We first observe that before the disease can be eradicated, the number of sick has to become low in comparison to the number of healthy individuals. When the number of sick individuals is low, the healthy individuals need to have a high risk aversion constant for their risk averseness to matter. In contrast, even small values of the empathy constant by the sick individuals can cause behavior change when the number of healthy individuals in the population is relatively high. Second, we observe that self-isolation decisions of sick individuals guarantee the prevention of disease spread to all their neighbors, whereas self-isolation decisions of a healthy individual averts only a single potential infection. It is a combination of these two observations that yield the effectiveness of empathy over risk aversion.

Previous models of behavior response to disease spread considered individual protective measures, e.g., social distancing, which are measures taken by healthy individuals^31^. Here we incorporated the response of the infected individuals as individuals willing to reduce their risk of transmitting to others. While we used the term self-isolation for the utmost measures taken both types (sick and healthy), these actions are based on different motives (empathy vs. risk aversion). The imbalance of roles played by the response of the infected versus the susceptible individuals in disease eradication affords critical policy insights. In particular, it suggests that public health recommendations should emphasize preventative practices when sick, e.g., covering cough, not going to work. It also highlights the importance of accounting for individual response in predictions of disease spread. It is worth noting that we can interpret the behavior response of infected individuals as altruism as termed and noted empirically^9^,^26^ since their worry is to refrain from infecting their neighbors.

This study focused on explaining the effects of individual measures on disease eradication. A future analytical focus of interest is to look at the combined effects of empathy and risk-aversion on longer-term effects when the disease is endemic. Further, in reality, these individual measures are coupled with policies implemented by public health institutions. As a future research direction, it would be interesting to couple the decentralized individual reactions to disease prevalence with centralized policies such as public information, isolating individuals that are sick, or vaccination campaigns. Another potential direction could be to modify the benchmark assumption that individuals behave rationally. Analysis of the sensitivity of the results to deviations from the rationality is of interest but it is not trivial how these behavioral deviations would be modeled^37^. Finally, the present model considered an SIS disease spread. It would be interesting to extend this model by including exposed individuals who are suspected of being infectious and the behavioral responses of the exposed type. Despite its simplifications, the current model provides a principled approach to connect rational decision making with other complex dynamic disease models. We are hopeful that further extensions will provide insights on how to influence the short- and long-term behavior of individuals so as to reduce the spread and burden of infectious disease.

## Methods

### Stochastic disease dynamics

We consider stochastic SIS disease dynamics where an individual *i* in the population 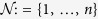 is either susceptible (*s*_*i*_(*t*) = 0) or infected (*s*_*i*_(*t*) = 1) at any given time 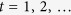. If the individual is susceptible at time *t*, it gets infected at time *t* + 1 with probability 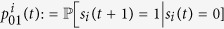. If the individual is sick at time *t*, it becomes susceptible at time *t* + 1 with probability 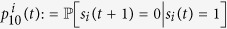. These transition probabilities define a Markov chain for the disease dynamics of individual 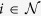 as follows


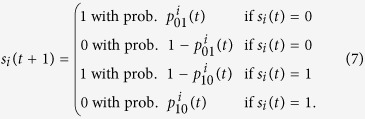


We denote the state of the disease in the population at time *t* by the vector of individual disease states, 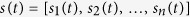.

A susceptible individual can only contract the disease in the next time step if in contact with an infected individual. We define the set of contacts of each individual by a contact network 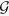 with node set 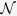 and edge set 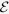 – see Fig. 1(a) for an example. The contact neighborhood of individual *i* is 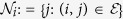. The chance of a susceptible individual (*s*_*i*_(*t*) = 0) contracting the disease from a neighboring infected contact (*s*_*j*_(*t*) = 1) depends on the infection probability of the disease 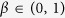, *i*’s action 0 ≤ *a*_*i*_(*t*) ≤ 1, and the contact’s action 0 ≤ *a*_*j*_(*t*) ≤ 1. In particular, the probability of 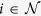 getting infected from individual 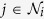 is equal to *βa*_*i*_(*t)a*_*j*_(*t)s*_*j*_(*t*). Assuming each local interaction is independent, we have


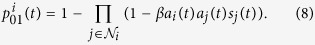


Each term inside the product is the probability that the individual *i* is not infected by neighbor *j*. The product of all the terms is the probability that the individual is not infected by any one of possible interactions. Finally, subtracting the product from one gives the probability that individual *i* contracts the disease.

The other important event in the SIS disease dynamics in (7) is the transition of an infected individual to a susceptible state which is equal to the inherent healing rate of the disease 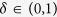, i.e., 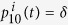 for 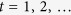.

The individual *i*’s action (*a*_*i*_(*t*)) represents preemptive measures, e.g., wearing a protective mask, or reducing social interaction, that the individual can take at time *t*, where *a*_*i*_(*t*) = 0 means *self-isolate* and *a*_*i*_(*t*) = 1 means resuming *normal* social interaction with no protective measures. If the actions of all individuals are equal to one, *a*_*i*_(*t*) = 1 for all times, then the model recovers the disease spread models over contact networks that do not include individual behavior response^14^. The Markov chain disease dynamics in (7) generalizes these models to include preemptive actions (*a*_*i*_(*t*) and *a*_*j*_(*t*)) as variables that affect infection probability 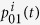 in (8).

### Individual preferences: bilinear payoffs

Individuals determine their actions based on their risk of getting infected or their potential to infect others. If individual *i* is susceptible (*s*_*i*_(*t*) = 0), individual *i* has a concern for contracting the disease proportional to the probability of infection 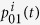 in the next time step. If individual *i* is infected (*s*_*i*_(*t*) = 1), individual *i* is concerned about infecting others in their neighborhood. This concern is proportional to the probability that *i* infects *j (βa*_*i*_*a*_*j*_) for 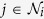. Finally, individual *i* has an incentive to avoid the cost of preemptive measures, i.e., continue normal levels of social interaction. A weighted linear combination of these preferences have the following form,


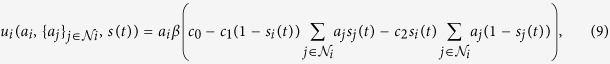


where *c*_0_, *c*_1_, *c*_2_ are positive constants. Inside the paranthesis, the first term is the socialization payoff, the second term is the cost of risk aversion that is nonzero only when individual *i* is susceptible (*s*_*i*_(*t*) = 0) and increases with the number of infected neighbors that do not take any preemptive measures, and the third term is the cost from the risk of infecting others that is nonzero only when individual *i* is infected (*s*_*i*_(*t*) = 1) and increases with the number of healthy neighbors that do not take any protective measures. We refer to *c*_0_, *c*_1_, and *c*_2_ as *socialization, risk averseness*, and *empathy* constants, respectively.

Note that the payoff above is a bilinear function of *a*_*i*_ and *a*_*j*_ for 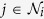. Maximizing the above utility function given 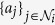 and *s(t*) with respect to individual *i*’s action *a*_*i*_, we obtain whether an individual resumes normal activity (*a*_*i*_ = 1) or self-isolates (*a*_*i*_ = 0) depending on the sign of expression inside the parantheses. In particular, if this expression is positive, *i* takes action *a*_*i*_ = 1. Otherwise, the action that maximizes the utility is to self-isolate.

In reality, the actions of neighboring individuals 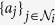 are not available to the individual. The payoffs of the neighbors of individual *i* depend on the actions of their own neighbors, i.e., the actions of other individuals as well as *i*. This means in a connected contact network 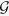, payoffs couple the actions of all the individuals. Hence, individuals need to reason about the interaction levels of their neighbors in their decision-making. We model individuals’ reasoning using game theory.

### Stochastic disease game and Myopic Markov Perfect Equilibrium (MMPE)

The stochastic process defined above, with the state transition dynamics in (7) and payoffs that depend only on the current state, is a stochastic game. The stochastic disease game is defined by the state variables 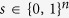, action spaces 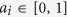, a transition function 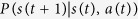 defined by equations in (7) that gives the probability of state at time *t* + 1 given the state *s(t*) and actions 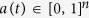 at time *t*, and the payoff functions of individuals *u*_*i*_(·) in (9). The game starts at an arbitrary disease state 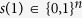. The following definition of rational behavior that we use in this paper assumes that individuals only react to the current state of the disease.

**Definition 1** *The strategy of individual i at time t, σ*_*i*_ *is a mapping from the state s(t) to the action space* [0, 1], *i.e*., 
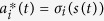
. *A myopic Markov perfect equilibrium strategy profile* 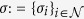 *is such that for all* 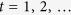, *the state s(t) and* 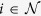 *it holds that*


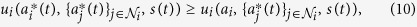


*for any* 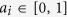 *and the state evolves according to the Markov chain in* (7).

The equilibrium definition given above implies that an MMPE strategy profile is stationary, that is, it only depends on the state. We defined the MMPE strategy profile such that each individual’s action at each stage is solely determined by the state of the disease. That is, individuals’ strategies are degenerate distributions on the action space, known as pure strategy equilibria^32^. In Supplementary Section A, we show that there exists at least one such strategy profile for the bilinear game in (9). Our proof of existence is constructive and it yields an algorithm that computes an MMPE strategy profile in finite time. This demonstrates that complexity of computing equilibrium behavior is low.

### Simulation details

Our simulations follow Markov chain dynamics. For single infected initializations, we randomly select an individual and run the simulation until the time horizon is reached or no infected individuals remain. We construct the scale-free contact network according to the preferential attachment algorithm^38^. At each step of the algorithm, we first solve for the equilibrium action of each individual to determine the transition probabilities of the Markov chain. In the following section we detail the process of solving the equilibrium action. Once the equilibrium actions are determined, we propagate the state of the disease by one time step according to the probabilities in (7). The code for our simulations is available online^39^.

### Solving for MMPE

For the game considered here, we propose a process that solves for the rational actions of individuals given a disease state 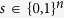. The details of the process are available at the Supplementary Section A. Here we provide an overview of the process that has two stages. The first stage entails iterated elimination of dominated actions. An action is dominated if there exists another action that the individual prefers in every possible circumstance. It is given that if an action is dominated it cannot be a rational action. For any game we can iteratively eliminate dominated actions.

For the disease network game considered here, the iterated elimination process starts with the action space 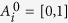 indicating all actions are possible. Next, we iterate 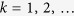 where at each step *k* we are given a set of individuals that have the remaining (not dominated) action space as 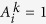 or 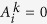. We denote the individuals that only have the socialize action 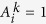 as the only not dominated action with 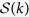. We denote the individuals that only have the self-isolate as the only not dominated action with 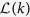. Given these individuals, we check for whether the following inequality is true for the remaining individuals 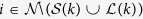 given disease state 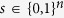,


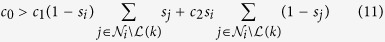


If this inequality is true, it means that individual *i* prefers to take action 1 regardless of what the neighbors of *i* that do not belong to the set 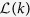, 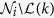, choose to do. Hence, all other actions of *i* are dominated by the socialize action. Individuals that satisfy the above inequality make up the set 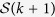 together with the individuals from previous iterations steps 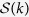.

At the same iteration step *k*, we check for the following inequality for the remaining individuals 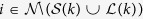 given disease state 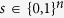,


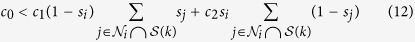


If this inequality is true, it means that individual *i* prefers to take action 0 even given their social neighbors belonging to the set 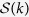. Hence, all other actions of *i* are dominated by the self-isolate action. Individuals that satisfy the above inequality make up the set 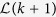 together with the individuals from previous iterations steps 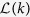.

In the Supplementary Section A we show that the process described above eliminates all dominated actions in at most *n* iteration steps where *n* is the population size. Note that at the end of the process if all individuals have a single not dominated action, i.e., 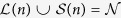, then it must be that it is the equilibrium action for the given disease state.

If there remains individuals that could not eliminate any actions, i.e., if 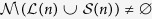, then we move to the second stage of the process. The second stage of the process assigns all infected remaining individuals to action *a*_*i*_ = 0 (*a*_*i*_ = 1) and all remaining susceptible individuals to action *a*_*i*_ = 1 (*a*_*i*_ = 0). We show in the Supplementary Section A, these two possible selections are indeed equilibria of the game.

The process outlined above is repeated at each time given the new state of the disease during our simulations. The code for this process is available online^39^.

## Additional Information

**How to cite this article:** Eksin, C. *et al*. Disease dynamics in a stochastic network game: a little empathy goes a long way in averting outbreaks. *Sci. Rep.* **7**, 44122; doi: 10.1038/srep44122 (2017).

**Publisher's note:** Springer Nature remains neutral with regard to jurisdictional claims in published maps and institutional affiliations.

## Supplementary Material

Supplementary Information
