# Supplementary material for: Disease dynamics in a stochastic network game: a little empathy goes a long way in averting outbreaks
Source: Sci Rep. 2017 Mar 14;7:44122. doi: 10.1038/srep44122 (PMC5349521; doi:10.1038/srep44122)
Supplement: Supplementary Information [file srep44122-s1.pdf]

Disease dynamics in a stochastic network game: a little empathy goes a long way in averting outbreaks

Supplementary Material

Ceyhun Eksin, Jeff S. Shamma and Joshua S. Weitz

A Existence of an MMPE strategy profile

We recall the utility function of individual i defined in the Methods section below,

$$u_i(a_i, \{a_j\}_{j\in\mathcal{N}_i}, s(t)) = a_i\beta\left(c_0 - c_1(1-s_i(t))\sum_{j\in\mathcal{N}_i} a_j s_j(t) - c_2 s_i(t)\sum_{j\in\mathcal{N}_i} a_j(1-s_j(t))\right). \tag{S1}$$

Define the space of probability distributions on the action space ($0 \le a_i \le 1$) $[0,1]$ as $\triangle([0,1])$. A mixed strategy profile $\sigma(\cdot)$ is a function that maps the state $s \in \{0,1\}^n$ to the space of probability distributions on the actions space, i.e., $\sigma_i : \{0,1\}^n \to \triangle([0,1])$. The definition of a mixed MMPE strategy profile $\sigma^* := \{\sigma_i^* : \{0,1\}^n \to \triangle[0,1]\}$ is a distribution on the action space that satisfies the following, for all $t = 1,2,\dots,$ and $i \in \mathcal{N}$,

$$u_i(\sigma_i^*(s(t)), \sigma_{\mathcal{N}_i}^*(s(t)), s(t)) \ge u_i(\sigma_i(s(t)), \sigma_{\mathcal{N}_i}^*(s(t)), s(t)) \tag{S2}$$

for any $\sigma_i \in \{0,1\}^n \to \triangle([0,1])$ where $\sigma_{\mathcal{N}_i}^* := \{\sigma_j^* : j \in \mathcal{N}_i\}$. From the existence of a mixed Nash equilibrium in games with continuous payoffs, we know that a mixed MMPE exists for the game with payoffs (S1).

In the following we constructively show that in the stochastic disease network game with payoffs in (S1), a degenerate (pure) MMPE strategy profile $\sigma^* := \{\sigma_i^* : \{0,1\}^n \to [0,1]\}$ that satisfies the above relation in (S2) exists. Note that a degenerate distribution puts weight one on a single action value, that is, the strategy profile corresponds to a single action profile for any state.

Since in the stochastic game population response is determined by the current state of the disease only, i.e., equilibrium is stationary, it suffices to show the existence of a pure Nash equilibrium strategy for the stage game with state $s \in \{0,1\}^n$. A pure Nash equilibrium (NE) strategy profile $\sigma^* : \{0,1\}^n \to [0,1]^n$ of the stage game with payoffs (S1) and state s satisfies

$$u_i(\sigma_i^*, \sigma_{\mathcal{N}_i}^*, s) \ge u_i(\sigma_i, \sigma_{\mathcal{N}_i}^*, s) \tag{S3}$$

for any $\sigma_i \in \{0,1\}^n \to \triangle([0,1])$. We define the corresponding equilibrium action profile as $a^* := \sigma^*(s)$ for a given state s.

For a given individual i with state $s_i \in \{0,1\}$, neighbors' state $s_{\mathcal{N}_i} := \{s_j\}_{j\in\mathcal{N}_i}$, and neighbor action profile $a_{\mathcal{N}_i}$ we have the best response of individual i as follows,

$$BR_i(a_{\mathcal{N}_i}, s_i, s_{\mathcal{N}_i}) := \underset{a_i\in[0,1]}{\operatorname{argmax}}\, u_i(a_i, a_{\mathcal{N}_i}, s_{\mathcal{N}_i}) \tag{S4}$$

$$= \mathbf{1}\left(c_0 > c_1(1-s_i)\sum_{j\in\mathcal{N}_i} a_j s_j + c_2 s_i \sum_{j\in\mathcal{N}_i} a_j(1-s_j)\right) \tag{S5}$$

where $\mathbf{1}(\cdot)$ is the indicator function. Since the payoffs are linear in self-actions, the actions that maximize the payoffs are in the extremes – $a_i = 1$ or $a_i = 0$ – depending on the states and actions of their neighbors. We can equivalently represent the NE definition in (S3) as a fixed point equation by using the best response definition,

$$a_i^* = BR_i(a_{\mathcal{N}_i}^*, s_i, s_{\mathcal{N}_i}) \quad \forall i \in \mathcal{N}. \tag{S6}$$

In the following we define the concept of strictly dominated action which will be a useful in finding the Nash equilibrium.

Definition 1 (Strictly dominated action) *For a given state $s \in \{0,1\}^n$, an action $a_i \in [0,1]$ is strictly dominated if and only if there exists an action $a_i' \in [0,1]$ such that*

$$u_i(a_i', a_{\mathcal{N}_i}, s) > u_i(a_i, a_{\mathcal{N}_i}, s) \quad \forall a_{\mathcal{N}_i} \tag{S7}$$

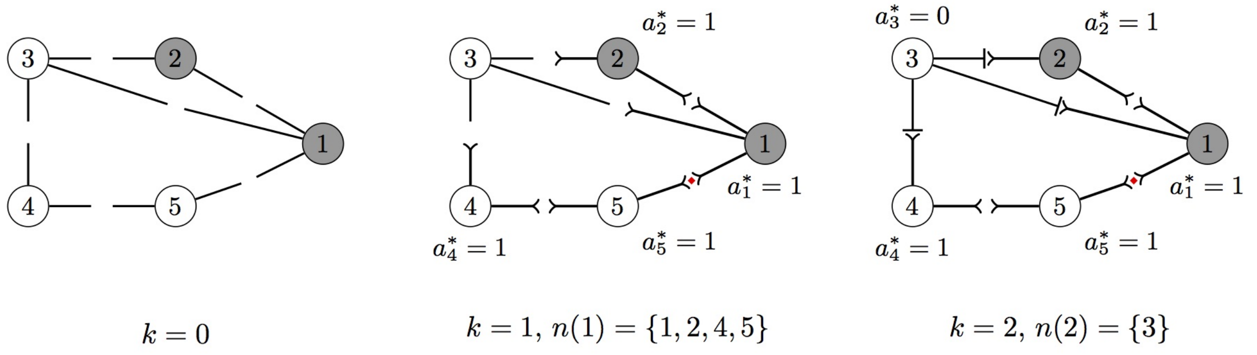

Figure S1. The iterated elimination process in Definition 3 on a 5 individual network. The payoff constants are such that $2c_1 > c_0 > c_1$ and $c_0 > 2c_2$. Individuals 1 and 2 are sick ($s_1 = 1$, $s_2 = 1$) and the rest are healthy. The contact network is shown at $k = 0$. At step $k = 1$, individuals in $n(1) = \{1,2,4,5\}$ eliminate all actions except 1 by (S9) given $c_0 > c_1$ and $c_0 > 2c_2$. Individual 3 cannot eliminate any action from the space. At step $k = 2$, individual 3 eliminates all actions except 0 by (S10). Since all individuals have singleton non-dominated action spaces, i.e., $\mathscr{N} \setminus (n(1) \bigcup n(2)) = \emptyset$, the process ends. Furthermore, the corresponding action profile is a Nash equilibrium of the stage game in (S1) by Lemma 2.

If an action a_i is strictly dominated then there exists a more preferable action a_i' for any circumstance. It is clear that if an action is strictly dominated then it cannot be a rational action from (S6). In a game we can iteratively remove the strictly dominated actions, this process is called the iterated elimination of strictly dominated strategies and is defined below.

Definition 2 (Iterated elimination of strictly dominated actions) *Set the initial set of actions $A_i^0 = [0,1]$ for all i, and for any $k \in \mathbb{N}$ set*

$$A_i^k = \{a_i \in A_i^{k-1} \,|\, a_i \text{ is not strictly dominated given any } a_{\mathscr{N}_i} \in A_{\mathscr{N}_i}^{k-1}\} \tag{S8}$$

We denote the set of actions of individual i that survive the iterated elimination by $A_i^\infty := \bigcap_{k=0}^{\infty} A_i^k$. When A_i^∞ has a single element, we say A_i^∞ is a singleton.

We formally state an iterated elimination process for the game in (S1).

Definition 3 (An iterated elimination process for the epidemic game) *Begin with action spaces $A_i^0 = [0,1]$ for all $i \in \mathscr{N}$. Fix $s \in \{0,1\}^n$. Iterate $k = 1,2,\dots$*

1) *[k = odd] Let $A_i^k = 1$ for all $i \in n(k)$ where*

$$n(k) := \left\{ i \in \mathscr{N} \setminus \bigcup_{l=1}^{k-1} n(l) : c_0 > \left(c_1(1-s_i) \sum_{j \in \mathscr{N}_i \setminus \bigcup_{l=even} n(l)} s_j + c_2 s_i \sum_{j \in \mathscr{N}_i \setminus \bigcup_{l=even} n(l)} (1-s_j) \right) \right\} \tag{S9}$$

2) *[k = even] Let $A_i^k = 0$ for all $i \in n(k)$ where*

$$n(k) := \left\{ i \in \mathscr{N} \setminus \bigcup_{l=1}^{k-1} n(l) : c_0 < \left(c_1(1-s_i) \sum_{j \in \mathscr{N}_i \bigcap \bigcup_{l=odd} n(l)} s_j + c_2 s_i \sum_{j \in \mathscr{N}_i \bigcap \bigcup_{l=odd} n(l)} (1-s_j) \right) \right\} \tag{S10}$$

If $n(k) = \emptyset$ then stop iteration of k.

Our next result shows that the process described above eliminates all strictly dominated strategies in finite time.

Lemma 1 *Consider the process described in Definition 3. There exists an iteration step $k \le n$ such that $\{A_i^k\}_{i \in \mathscr{N}}$ are the set of actions that are not strictly dominated for the game with payoffs (S1) and state $s \in \{0,1\}^n$.*

Proof: First consider the odd iteration steps, $k = odd$. If the condition inside the bracket in (S9) holds then for any action that individual $j \in \mathscr{N}_i \setminus \bigcup_{l=even} n(l)$ takes, $a_i' = 1$ dominates any other action $a_i \in [0,1] \setminus \{1\}$ by Definition 1. To see this first

recall the best response of individual i (S5). Note that by the even steps, individuals $j \in \bigcup_{l=even} n(l)$ have $a_j = 0$ as the only not dominated action. Hence the best response of individual i can be rewritten as

$$\mathbf{1}\left(c_0 > \left(c_1(1-s_i)\sum_{j\in\mathcal{N}_i\setminus\bigcup_{l=even}n(l)} a_j s_j + c_2 s_i \sum_{j\in\mathcal{N}_i\setminus\bigcup_{l=even}n(l)} a_j(1-s_j)\right)\right) \tag{S11}$$

where we remove neighbors that only have zero as the not dominated action. If the inequality inside the indicator function is true even when the remaining neighbors of i take action 1, then $a_i = 1$ dominates all the other actions $[0,1)$ of i, that is, for all $a_j \in [0,1]$ for $j \in \mathcal{N}\setminus i$,

$$\mathbf{1}\left(c_0 > \left(c_1(1-s_i)\sum_{j\in\mathcal{N}_i\setminus\bigcup_{l=even}n(l)} s_j + c_2 s_i \sum_{j\in\mathcal{N}_i\setminus\bigcup_{l=even}n(l)} (1-s_j)\right)\right) \leq \mathbf{1}\left(c_0 > \left(c_1(1-s_i)\sum_{j\in\mathcal{N}_i\setminus\bigcup_{l=even}n(l)} a_j s_j + c_2 s_i \sum_{j\in\mathcal{N}_i\setminus\bigcup_{l=even}n(l)} a_j(1-s_j)\right)\right) \tag{S12}$$

Assuming the left hand side of the above inequality is one then by the definition of strictly dominated action in Definition 1, $a_i = 1$ is the only action that is not dominated.

Next, we consider the even iteration steps, $k = even$. If the condition inside the bracket in (S10) holds then for any action that individual $j \in \mathcal{N}_i \setminus \bigcup_{l=odd} n(l)$ takes, $a'_i = 0$ dominates $a_i \in [0,1]\setminus\{0\}$ by Definition 1. To see this first recall the best response of individual i (S5). Note that by the odd steps, individuals $j \in \bigcup_{l=odd} n(l)$ have $a_j = 1$ as the only not dominated action. Hence the best response of individual i can be rewritten as

$$\mathbf{1}\left(c_0 > \left(c_1(1-s_i)\Big(\sum_{j\in\mathcal{N}_i\setminus\bigcup_{l=odd}n(l)} a_j s_j + \sum_{j\in\mathcal{N}_i\bigcap\bigcup_{l=odd}n(l)} s_j\Big) + c_2 s_i\Big(\sum_{j\in\mathcal{N}_i\setminus\bigcup_{l=odd}n(l)} a_j(1-s_j) + \sum_{j\in\mathcal{N}_i\bigcap\bigcup_{l=odd}n(l)} (1-s_j)\Big)\right)\right) \tag{S13}$$

where we separate neighbors $j \in \mathcal{N}_i \bigcap \bigcup_{l=odd} n(l)$ that only have $a_j = 1$ as the not dominated action from the other neighbors $j \in \mathcal{N}_i \setminus \bigcup_{l=odd} n(l)$. If the inequality inside the indicator function is false even when the remaining neighbors of i take action 0, then $a_i = 0$ dominates all the other actions $(0,1]$ of i, that is, for any $a_j \in [0,1]$ for $j \in \mathcal{N}_i \setminus \bigcup_{l=odd} n(l)$

$$\mathbf{1}\left(c_0 < \left(c_1(1-s_i)\Big(\sum_{j\in\mathcal{N}_i\bigcap\bigcup_{l=odd}n(l)} s_j\Big) + c_2 s_i\Big(\sum_{j\in\mathcal{N}_i\bigcap\bigcup_{l=odd}n(l)} (1-s_j)\Big)\right)\right) \leq \mathbf{1}\left(c_0 < \left(c_1(1-s_i)\Big(\sum_{j\in\mathcal{N}_i\setminus\bigcup_{l=odd}n(l)} a_j s_j + \sum_{j\in\mathcal{N}_i\bigcap\bigcup_{l=odd}n(l)} s_j\Big) + c_2 s_i\Big(\sum_{j\in\mathcal{N}_i\setminus\bigcup_{l=odd}n(l)} a_j(1-s_j) + \sum_{j\in\mathcal{N}_i\bigcap\bigcup_{l=odd}n(l)} (1-s_j)\Big)\right)\right) \tag{S14}$$

Assuming the left hand side of the above inequality is one then by the definition of strictly dominated action in Definition 1, $a_i = 0$ is the only action that is not dominated.

Further if no individual can eliminate a dominated strategy $n(k) = \emptyset$ at an iteration k then at following iterations $k+1, k+2, \ldots$ there won't be any individuals that eliminate any actions as strictly dominated. To see this, assume the opposite is true, that is, $n(k+1) \neq \emptyset$. Since $n(k) = \emptyset$ then the condition in $n(k+1)$ is identical to the conditions inside (S9) or (S10) for $n(k-1)$ depending on whether $k+1$ is odd or even, respectively. There cannot be an individual that satisfies the conditions at iteration $k+1$ because $n(k+1)$ is selected among individuals that have not been previously in any set, i.e., $i \in \mathcal{N} \setminus \bigcup_{l=1}^{k} n(l)$. This contradicts $n(k+1) \neq \emptyset$. As a result, at each iteration k the number of individuals in $n(k)$ has to be positive until an iteration step $n(k)$ at which either there is no individual left $\mathcal{N} \setminus \bigcup_{l=1}^{k} n(l) = \emptyset$ or there is no individual that satisfies the condition after the colon in (S9) or (S10).

Suppose now that the process stops at iteration k, i.e., $n(k) = \emptyset$, but there exists an individual $i \in \mathcal{N} \setminus \bigcup_{l=1}^{k-1} n(l)$ with strictly dominated action (S7) $a_i \in A_i^k$. Suppose k is odd then by (S12), any action $a_i \in A_i^k \setminus \{1\}$ must be dominated by $a'_i = 1$. Suppose k is even, then by (S14) any action $a_i \in A_i^k \setminus \{0\}$ must be dominated by $a'_i = 0$. Then $n(k) \neq \emptyset$ which is a contradiction. This

together with the fact that if $n(k)=\emptyset$ then $n(l)=\emptyset$ for $l>k$ implies that if the process in Definition 3 stops all the strictly dominated strategies are eliminated.

Finally, the fact that the number of elements of $n(k)$ is positive at every iteration except the last iteration in Definition 3 implies that the iteration ends in at most n iterations.

The following Lemma shows that once all the strictly dominated strategies are eliminated, the action profile that assigns socialize to susceptible individuals and do not socialize to infected individuals is a pure Nash equilibrium strategy profile.

Lemma 2 *Consider the game defined by the payoffs in (S1) given state $s\in\{0,1\}^n$ and the iterated elimination process in Definition 3. Denote the action space of $i\in\mathcal{N}$ that is not strictly dominated by A_i^*. Let $a_i^*=A_i^*$ if A_i^* is a singleton. If A_i^* is not a singleton then let $a_i^*=1$ for $s_i=0$ and $a_i^*=0$ for $s_i=1$. The resulting action profile a^* is a pure Nash equilibrium of the game.*

Proof: *We will use the following equivalent definition in (S6) of Nash equilibrium for the stage game in (S1),*

$$a_i^* = \mathbf{1}\left(c_0 > c_1(1-s_i)\sum_{j\in\mathcal{N}_i} a_j^* s_j + c_2 s_i \sum_{j\in\mathcal{N}_i} a_j^*(1-s_j)\right). \tag{S15}$$

Note that at the end of the elimination process in Definition 3, the action space of an individual i is either a singleton or is equal to $A_i^*=[0,1]$. A strictly dominated action cannot satisfy the above equation, hence the equilibrium action of an individual with a singleton non-dominated action space is given by $a_i^*=A_i^*$.

Now consider $i\in\mathcal{N}$ such that $A_i^*=[0,1]$. Suppose $a_i^*=1$ for a susceptible individual ($s_i=0$) is not a Nash equilibrium action. Then individual i can deviate and by the above equation it must be that $a_i^*=0$ is a Nash equilibrium action because

$$c_0 < c_1 \sum_{j\in\mathcal{N}_i} a_j^* s_j. \tag{S16}$$

Note that by our assumption $a_j^*=0$ for all the infected individuals ($s_j=1$) at which 0 is not a strictly dominated action, i.e., when $A_j=[0,1]$. Hence in the right hand side of the above inequality only individuals that do not have 0 in their not dominated action set will matter. Furthermore, if 0 is a strictly dominated action, socialize action 1 is the only remaining not strictly dominated action. Hence, we can write the above inequality as follows,

$$c_0 < c_1 \sum_{j\in\mathcal{N}_i:\{0\}\notin A_j^*} s_j. \tag{S17}$$

Now note that if the above inequality is true then 1 should be strictly dominated by action 0 for individual i. Hence, it is a contradiction to the fact that the action space is not a singleton, $A_i^*\in[0,1]$.

A similar contradiction argument can be made for the infected individuals ($s_i=1$) and the equilibrium action $a_i^*=0$. Therefore, the action profile described in the statement must be a pure Nash equilibrium of the game.

Lemma 1 shows that the process in Definition 3 eliminates all dominated actions in finite time. Furthermore, if all individuals are included in the process, i.e., if $\mathcal{N}=\bigcup_{k=1}^n n(k)$ then we end up with a singleton action profile that is not strictly dominated. This means the game has an unique pure Nash equilibrium by definition of strictly dominated action. If at the end of the process in Definition 3, if all individuals are not included in the process, i.e., $\mathcal{N}\setminus\bigcup_{k=1}^n n(k)\neq\emptyset$ then the set of not strictly dominated actions is not a singleton. Lemma 2 proposes a pure strategy profile that is a Nash equilibrium of the game in (S1) for the case that action spaces of individuals that survive strict elimination process are not all singleton.

Lemmas 1 and 2 considered the stage game with payoffs (S1) given state $s\in\{0,1\}^n$. An MMPE strategy profile σ is a mapping from any state to the action space. We can obtain a pure MMPE strategy profile using Lemma 2 for all possible states $s\in\{0,1\}^n$. That is, we use the elimination process in Definition 3 and the action assignment given in Lemma 2 for all the states to construct an MMPE strategy profile. In our simulations, in this paper, we construct the MMPE equilibrium strategy profiles following this process – see Fig. S1 for an example.

B Price of Anarchy of the stage game

In this section, we consider the sub-optimality of the decisions of individuals that play according to an MMPE strategy profile. By the definition of MMPE strategy, individuals consider current payoffs and play according Nash equilibrium strategy of that stage game. In the following we show the worst stage game Nash equilibrium strategy can be n fold worse than the optimal action profile given a network and state.

Define the stage game $\Gamma(s,\mathscr{G},c)$ with payoffs in (S1) parametrized by the disease state s, contact network $\mathscr{G}$ and utility constants $c := \{c_0, c_1, c_2\}$. The welfare value of the action profile a in this game is the sum of utilities of the individuals,

$$W(a,s(t)) = \sum_{i=1}^{n} u_i(a_i, a_{\mathscr{N}_i}, s(t)) = \sum_{i=1}^{n} c_0 a_i - (c_1 + c_2) \sum_{(i,j)\in\mathscr{E}} (1 - s_i(t)) s_j(t) a_i a_j. \tag{S18}$$

Note that the first summation is over all the individuals and the second summation is over all the edges $\mathscr{E}$ in the network $\mathscr{G}$. We define the optimum action profile as the maximizer of the welfare function above, i.e., $a^{opt} = \operatorname{argmax}_a W(a,s(t))$. We denote the set of Nash equilibrium action profiles by A^* and define the price of anarchy (PoA) as the ratio of the worst possible Nash action profile to the optimum action profile,

$$PoA := \frac{\min_{a\in A^*} W(a)}{W(a^{opt})}. \tag{S19}$$

Obviously, $PoA \leq 1$. In the following we provide a lower bound on the price of anarchy.

Proposition 1 *For a game $\Gamma(s,\mathscr{G},c)$, the price of anarchy (S19) has the following lower bound*

$$PoA \geq 1 - \frac{\max_{i\in\mathscr{N}} |\mathscr{N}_i| \max(c_1, c_2)}{n c_0} \tag{S20}$$

where $|\mathscr{N}_i|$ is the degree connectivity of i.

Proof: *Consider the derivatives of u_i in (S1) and W in (S18) with respect to a_i, respectively,*

$$\frac{\partial u_i}{\partial a_i} = c_0 - \left(c_1 (1 - s_i) \sum_{j\in\mathscr{N}_i} s_j a_j + c_2 s_i \sum_{j\in\mathscr{N}_i} (1 - s_j) a_j \right) \tag{S21}$$

$$\frac{\partial W}{\partial a_i} = c_0 - (c_1 + c_2) \left((1 - s_i) \sum_{j\in\mathscr{N}_i} s_j a_j + s_i \sum_{j\in\mathscr{N}_i} (1 - s_j) a_j \right). \tag{S22}$$

First note that $\frac{\partial u_i}{\partial a_i} \geq \frac{\partial W}{\partial a_i}$ for any s and a_{-i} because

$$\frac{\partial W}{\partial a_i} = \frac{\partial u_i}{\partial a_i} - \left(c_2 (1 - s_i) \sum_{j\in\mathscr{N}_i} s_j a_j + c_1 s_i \sum_{j\in\mathscr{N}_i} (1 - s_j) a_j \right). \tag{S23}$$

Therefore, given actions of others a_{-i} and state s, it could be that $\frac{\partial u_i}{\partial a_i} > 0$ while $\frac{\partial W}{\partial a_i} < 0$. Consider the optimal and equilibrium action profiles where $a_j^{opt} = a_j^*$ for $j \in \mathscr{N} \setminus i$, and $a_i^{opt} = 0$ but $a_i^* = 1$. That is, we have $\frac{\partial u_j}{\partial a_j} < 0$ and $\frac{\partial W}{\partial a_j} < 0$ for all $j \neq i$ but $\frac{\partial u_i}{\partial a_i} > 0$ while $\frac{\partial W}{\partial a_i} < 0$. Then, the difference between the equilibrium profile welfare $W(a^*,s)$ and optimal action profile welfare $W(a^{opt},s)$ is equal to (S23), that is,

$$W(a^*,s) - W(a^{opt},s) = \frac{\partial W}{\partial a_i} \tag{S24}$$

We divide the difference above by $W(a^{opt},s)$ to get

$$-1 + \frac{W(a^*,s)}{W(a^{opt},s)} = \frac{\frac{\partial W}{\partial a_i}}{W(a^{opt},s)}. \tag{S25}$$

Now assume $\frac{\partial u_i}{\partial a_i} = \varepsilon > 0$ to get

$$\frac{W(a^*,s)}{W(a^{opt},s)} = 1 + \frac{\varepsilon - \left(c_2 (1 - s_i) \sum_{j\in\mathscr{N}_i} s_j a_j + c_1 s_i \sum_{j\in\mathscr{N}_i} (1 - s_j) a_j \right)}{W(a^{opt},s)}. \tag{S26}$$

A trivial lower bound on the term for the numerator above is $-\max(c_1,c_2)|\mathscr{N}_i|$ for any state s. Furthermore a trivial upper bound for $W(a^{opt},s)$ is nc_0. Using these bounds we obtain a bound for the above equality,

$$\frac{W(a^*,s)}{W(a^{opt},s)} \geq 1 - \frac{\max(c_1,c_2)|\mathscr{N}_i|}{n c_0} \tag{S27}$$

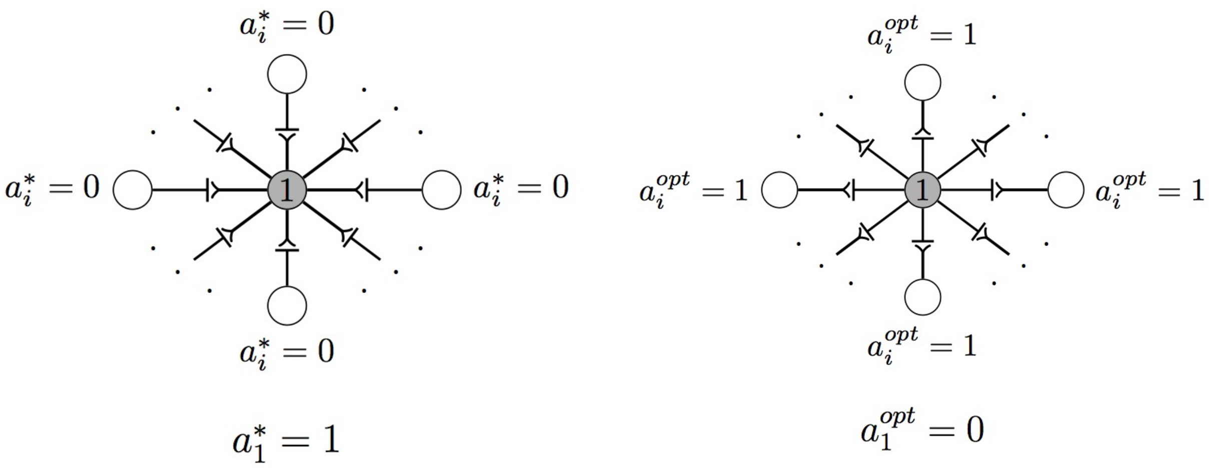

Figure S2. Price of anarchy in the star network (Example 1). We consider the strong empathy ($c_0 < (n-1)c_2$) and strong averseness ($c_0 < c_1$) case in Figure 2(d) of the main manuscript. Left and right figures show the two stage game pure Nash equilibria. The Nash equilibrium action profile shown by the right figure is optimal with welfare equal to $(n-1)c_0$. The Nash equilibrium action profile shown by the left figure attains a welfare equal to c_0. The PoA is equal to $1/n-1$.

Now note that the choice of the individual i is arbitrary. When this individual is the individual with the largest number of neighbors we get a lower bound on the right hand side of the above inequality which is the worst case lower bound on the price of anarchy in (S20).

Note that this upper bound can be arbitrarily bad, i.e., in the order of $1/n$. In the discussion following Figure 2(d) in the main text we show that this bound is tight. We repeat this example below.

Example 1 *Consider a star network with n individuals. The center individual with $n-1$ neighbors is the only infected individual. Payoff constants are such that $c_0 < c_1$ and $c_0 < (n-1)c_2$. There are two stage game Nash equilibria: 1) $a_i^{opt} = 1$ if $s_i = 0$ and $a_i^{opt} = 0$ if $s_i = 1$, and 2) $a_i^* = 0$ if $s_i = 0$ and $a_i^* = 1$ if $s_i = 1$. The first Nash equilibrium is also the optimal action profile a^{opt} yielding a welfare of $(n-1)c_0$. The second Nash equilibrium a^* obtains a welfare of c_0 resulting in a PoA of $\frac{1}{n-1}$.*

Another example on a star network follows.

Example 2 *Consider the star network shown in Figure S2. The center individual 1 has $n-1$ neighbors and is the only infected individual. Constants are such that $c_0 = c_1 + \rho_1$ and $c_0 = (n-1)c_2 + \rho_2$ for arbitrarily small positive constants $\rho_1 > 0$ and $\rho_2 > 0$, so that $c_0 < (n-1)(c_1 + c_2)$. The unique NE action profile is that all individuals are social, $a_i^* = 1$ for all $i \in \mathcal{N}$. The welfare for the NE is $W(a^*) = nc_0 - (n-1)(c_1 + c_2) = (n-1)\rho_1 + \rho_2$. The optimal action profile is that only the susceptible nodes are social $a_i^{opt} = 1$ for $i \neq 1$ and $a_1^{opt} = 0$. The welfare for the a^{opt} is $W(a^{opt}) = (n-1)c_0$. Then PoA $\approx \rho_1/c_0$ for large n.*

Here, we see that PoA is determined by the closeness of c_1 to c_0. Note that in this example, equilibrium is unique and, unlike Example 1, the optimal action profile is not a Nash equilibrium.

Another notion that enables us to gauge the optimality of equilibria is the Price of Stability (PoS). PoS is the ratio of the best possible Nash action profile to the optimum action profile,

$$PoS := \frac{\max_{a \in A^*} W(a)}{W(a^{opt})} \tag{S28}$$

Note that $PoS \leq 1$ by definition of a^{opt}. We remark that in Example 2, the equilibrium is unique, meaning $PoS = PoA$. Therefore, not only the PoA but also the PoS can be arbitrarily bad.

C R_0 bound

We formally define the reproductive ratio R_0 in the following.

Definition 4 *Let the initial state of the population be given by $s(1)$ where $s_i(1)=1$, otherwise $s_j(1)=0$ for all $j\neq i$ for some randomly selected individual i. Then R_0 is the expected number of individuals that contract the disease from the randomly selected individual i until i heals,*

$$R_0 := E\left[E\left[\sum_{t=1}^{\infty}\sum_{j=1}^{n}\mathbf{1}(s_j(t+1)-s_j(t)=1, i\to j)\mathbf{1}\left(s_i(l)=1 \text{ for } l<t\right)\,\middle|\,s(1)\right]\right] \tag{S29}$$

where $\mathbf{1}(s_j(t+1)-s_j(t)=1,i\to j)$ is the indicator function that is one if individual j transitions to an infected state at time t and i is the one infecting j, and $\mathbf{1}\left(s_i(l)=1 \text{ for } l<t\right)$ is the indicator function that is one if individual i has not healed yet. The outside expectation is with respect to the uniform distribution that selects the initial infected individual, and the inside expectation is with respect to the transition probabilities of the Markov chain.

In the following, we derive bounds for R_0 given that individuals act according to an MMPE strategy profile and we select the initial infected individual randomly from the network.

Theorem 1 *Consider a network with degree distribution $P(k)$. Assume the infected individual is chosen from the population uniformly at random. Scale c_0 with β for convenience, i.e., $c_0 := \beta c_0$, and assume $c_0 > c_1$.Assume $c_0 > c_1$. Then R_0 defined in (S29) has the following upper bound,*

$$R_0 \le \frac{\beta}{\delta}\sum_{k=1}^{K} kP(k) \tag{S30}$$

where $K := \min(\lfloor c_0/c_2\rfloor, n)$.

Proof: *We start by moving the second expectation inside the sum in the definition of R_0 (S29) to get the following,*

$$R_0 = E\left[\sum_{t=1}^{\infty}\sum_{j=1}^{n} E\left[\mathbf{1}(s_j(t+1)-s_j(t)=1,i\to j)\mathbf{1}\left(s_i(l)=1 \text{ for } l<t\right)\,\middle|\,s(1)\right]\right]. \tag{S31}$$

Now consider the conditional expectation inside the summation which we can equivalently represent as the following conditional probability

$$\begin{aligned} &E[\mathbf{1}(s_j(t+1)-s_j(t)=1,i\to j)\mathbf{1}\left(s_i(l)=1 \text{ for } l<t\right)\,|\,s(1)] \\ &\qquad = P\left(s_j(t+1)-s_j(t)=1, i\to j, s_i(l)=1 \text{ for } l<t \,\middle|\, s(1)\right). \end{aligned} \tag{S32}$$

Note that the above conditional probability is the probability that j is infected by i at time t and i remained infected until time t given i is infected at $t=0$. Using the chain rule and law of total probability, we can write the above conditional probability as follows

$$\begin{aligned} &P(s_j(t+1)-s_j(t)=1,i\to j,s_i(l)=1 \text{ for } l<t\,|\,s(1)) = \\ &\Big(P\left(s_j(t+1)-s_j(t)=1,i\to j\,\middle|\,s_i(t)=1,s_j(t)=0,s(1)\right)P(s_i(t)=1,s_j(t)=0\,|\,s_i(l)=1 \text{ for } l<t,s(1)) \\ &+P\left(s_j(t+1)-s_j(t)=1,i\to j\,\middle|\,s_i(t)=0,s_j(t)=0,s(1)\right)P(s_i(t)=0,s_j(t)=0\,|\,s_i(l)=1 \text{ for } l<t,s(1)) \\ &+P\left(s_j(t+1)-s_j(t)=1,i\to j\,\middle|\,s_i(t)=1,s_j(t)=1,s(1)\right)P(s_i(t)=1,s_j(t)=1\,|\,s_i(l)=1 \text{ for } l<t,s(1)) \\ &+P\left(s_j(t+1)-s_j(t)=1,i\to j\,\middle|\,s_i(t)=0,s_j(t)=1,s(1)\right)P(s_i(t)=0,s_j(t)=1\,|\,s_i(l)=1 \text{ for } l<t,s(1))\Big) \\ &P\left(s_i(l)=1 \text{ for } l<t\,\middle|\,s(1)\right) \end{aligned} \tag{S33}$$

Note that the first four lines on the right hand side equal to the probability that i infects j at time t given that i remained infected until $t-1$ by law of total probability. The last line is the probability that i remained infected given that i started infected since $s_i(1)=1$. Observe that the probability that i infects j is zero if individual i is susceptible at time t or individual j is infected, i.e., $P\left(s_j(t+1)-s_j(t)=1,i\to j\,\middle|\,s_i(t)=0\right)=0$ or $P\left(s_j(t+1)-s_j(t)=1,i\to j\,\middle|\,s_j(t)=1\right)=0$. Hence only the first line of the four line expression is nonzero which simplifies the identity above as follows

$$\begin{aligned} &P(s_j(t+1)-s_j(t)=1,i\to j,s_i(l)=1 \text{ for } l<t\,|\,s(1)) = \\ &\quad P\left(s_j(t+1)-s_j(t)=1,i\to j\,\middle|\,s_i(t)=1,s_j(t)=0,s(1)\right)P(s_i(t)=1,s_j(t)=0\,|\,s_i(l)=1 \text{ for } l<t,s(1)) \\ &\quad P\left(s_i(l)=1 \text{ for } l<t\,\middle|\,s(1)\right) \end{aligned} \tag{S34}$$

The probability of healing at each step is independent, hence the last conditional probability is equal to $(1-\delta)^{t-1}$. The conditional probability that i remains infected and j is infected at time t given that i is infected until time t is less than $1-\delta$ by the argument below,

$$\begin{aligned}
&P(s_i(t)=1, s_j(t)=0 \,|\, s_i(l)=1 \text{ for } l<t, s(1)) \\
&\quad = P(s_j(t)=0 \,|\, s_i(t)=1, s_i(l)=1 \text{ for } l<t, s(1)) P(s_i(t)=1 \,|\, s_i(l)=1 \text{ for } l<t, s(1)) && \text{(S35)}\\
&\quad \le P(s_i(t)=1 \,|\, s_i(l)=1 \text{ for } l<t, s(1)) && \text{(S36)}\\
&\quad = 1-\delta. && \text{(S37)}
\end{aligned}$$

The first equality above follows by chain rule. The inequality follows by the fact that the probability is less than one. The second equality is true by the transition probability of individual i from an infected state. Substituting these identities inside the equation (S34) we obtain the following

$$\begin{aligned}
&P(s_j(t+1)-s_j(t)=1, i\to j, s_i(l)=1 \text{ for } l<t \,|\, s(1)) \le \\
&\qquad P\left(s_j(t+1)-s_j(t)=1, i\to j \,\middle|\, s_i(t)=1, s_j(t)=0, s(1)\right)(1-\delta)^t. && \text{(S38)}
\end{aligned}$$

Now consider the conditional probability on the right hand side of (S38), the probability that j is infected at time $t+1$ by i given that j is susceptible and i is infected at time t. We have the following upper bound,

$$\begin{aligned}
&P(s_j(t+1)-s_j(t)=1, i\to j \,|\, s_i(t)=1, s_j(t)=0, s(1)) \\
&\qquad = P\left(s_j(t+1)=1, i\to j \,\middle|\, s_i(t)=1, s_j(t)=0, s(1)\right) \\
&\qquad = P\left(s_j(t+1)=1, i\to j \,\middle|\, a_i^*(t)=1, a_j^*(t)=1, s_i(t)=1, s_j(t)=0, s(1)\right) \\
&\qquad\quad P\left(a_i^*(t)=1, a_j^*(t)=1 \,\middle|\, s_i(t)=1, s_j(t)=0, s(1)\right) \\
&\qquad \le P\left(s_j(t+1)=1, i\to j \,\middle|\, a_i^*(t)=1, a_j^*(t)=1, s_i(t)=1, s_j(t)=0, s(1)\right) \\
&\qquad\quad P\left(a_i^*(t)=1 \,\middle|\, s_i(t)=1, s_j(t)=0, s(1)\right) \\
&\qquad = \beta \mathbf{1}(j\in\mathcal{N}_i) P\left(a_i^*(t)=1 \,\middle|\, s_i(t)=1, s_j(t)=0, s(1)\right) && \text{(S39)}
\end{aligned}$$

The first equality is by the fact that $s_j(t+1)=1$ if $s_j(t)=0$ and $s_j(t+1)-s_j(t)=1$. The second equality above is by the law of total probability and by the fact that if i or j takes an action to self-isolate, i.e., $a_j(t)=0$ or $a_i(t)=0$, then i cannot infect j. The inequality follows by the fact that $P\left(a_i(t)=1, a_j(t)=1 \,\middle|\, s_i(t)=1, s_j(t)=0\right) \le P\left(a_i(t)=1 \,\middle|\, s_i(t)=1, s_j(t)=0\right)$. The last equality follows because if both agents socialize at normal levels then the infection probability is β when agent j and i are connected, $j\in\mathcal{N}_i$.

Now we consider $a_i^*(t)$. We know that the MMPE action of individual i is a best response to best response actions of neighbors from (S6) where best response function is given by (S5). Specifically from the perspective of an infected individual i, the best response action is given by the following

$$a_i^*(t) = \mathbf{1}\left(c_0 > c_2 \sum_{j\in\mathcal{N}_i} a_j^*(t)(1-s_j(t))\right) \tag{S40}$$

Consider $t=1$. If $c_0>c_1$, then $a_j^*(1)=1$ for all $j\in\mathcal{N}_i$. Hence, if $c_0>c_2|\mathcal{N}_i|$ then $a_i^*(1)=1$, i.e., $a_i^*(1)=\mathbf{1}(c_0>c_2|\mathcal{N}_i|)$. Also note that if $a_i^*(1)=1$ then $a_i^*(t)=1$ for all $t>1$. Moreover, if $a_i^*(1)=0$ then i never infects another node if it is the only initially infected individual. Hence, we have $a_i^*(t)=a_i^*(1)$ for all t. That is, the action of agent i at time t is determined by the initial action and is independent of the state at time t. Therefore, we can write

$$\begin{aligned}
P\left(a_i^*(t)=1 \,\middle|\, s_i(t)=1, s_j(t)=0, s(1)\right) &= P\left(a_i^*(1)=1 \,\middle|\, s(1)\right) \\
&= \mathbf{1}(c_0>c_2|\mathcal{N}_i|). && \text{(S41)}
\end{aligned}$$

Substituting the above identity in (S39) and using (S39) in (S38), which then we substitute to (S32), we get the following upper bound for R_0 in (S31),

$$R_0 \le E\left[\sum_{t=1}^{\infty}\sum_{j=1}^{n} \beta \mathbf{1}(c_0>c_2|\mathcal{N}_i|)\mathbf{1}(j\in\mathcal{N}_i)(1-\delta)^t\right]. \tag{S42}$$

Now note the indicator function terms depend on the network of individual i but they do not depend on the identities of the neighbors. In fact, $\sum_{j=1}^{n} \mathbf{1}(j \in \mathcal{N}_i)$ is equal to the number of neighbors of i, i.e., $|\mathcal{N}_i|$. In addition, we have $\sum_{t=1}^{\infty}(1-\delta)^t = 1/\delta$. Using these identities, we can write the above bound as follows,

$$R_0 \leq \frac{\beta}{\delta} E\left[|\mathcal{N}_i| \mathbf{1}(c_0 > c_2|\mathcal{N}_i|)\right] \tag{S43}$$

Individual i is chosen uniformly random and it has k neighbors with probability $P(k)$, therefore, the expectation on the right hand side is equal to the following,

$$R_0 \leq \frac{\beta}{\delta} \sum_{k=1}^{n} k \mathbf{1}(k < \frac{c_0}{c_2}) P(k) \tag{S44}$$

Bound in (S30) follows.

D R_0 bound scale-free

Corollary 1 *Consider a random scale free network where the degree distribution follows $P(k) \sim k^{-\gamma}$ for $\gamma = 2$. Then R_0 defined in (S29) has the following upper bound,*

$$R_0 \leq \frac{n}{2n-1} \frac{\beta}{\delta} \log(\min(\lfloor c_0/c_2 \rfloor, n) + 1). \tag{S45}$$

Proof: *We directly use the bound in (S30) and substitute in $P(k) = L(-2,n)k^{-2}$ where $L(-2,n) = (\sum_{k=1}^{n} k^{-2})^{-1}$ is the normalization constant for the scale-free distribution*

$$R_0 \leq L(-2,n) \frac{\beta}{\delta} \sum_{k=1}^{\min(\lfloor c_0/c_2 \rfloor, n)} k^{-1} \tag{S46}$$

We first upper bound the normalization constant $L(-2,n)$ by the fact that $\sum_{k=1}^{n} k^{-2} > 2 - \frac{1}{n}$. Hence, $L(-2,n) \leq \frac{n}{2n-1}$. Next we note that the summation behaves logarithmically which yields the desired bound in (S45).

E R_* bound

We formally define the reproductive ratio R_* in the following.

Definition 5 *Let the initial state of the population be given by $s(1)$ where $s_i(1) = 1$, otherwise $s_j(1) = 0$ for all $j \neq i$ for some randomly selected individual i. The probability that we select an initial sick individual with degree k is given by $Q(k) := \frac{kP(k)}{\sum_k kP(k)}$. Then R_* is the expected number of individuals that contract the disease from a randomly selected individual i until i heals,*

$$R_* := E\left[E\left[\sum_{t=1}^{\infty}\sum_{j=1}^{n} \mathbf{1}(s_j(t+1) - s_j(t) = 1, i \to j) \mathbf{1}\left(s_i(l) = 1 \text{ for } l < t\right) \Big| s(1)\right]\right] \tag{S47}$$

where $\mathbf{1}(s_j(t+1) - s_j(t) = 1, i \to j)$ is the indicator function that is one if individual j transitions to an infected state at time t and i is the one infecting j, and $\mathbf{1}(s_i(l) = 1 \text{ for } l < t)$ is the indicator function that is one if individual i has not healed yet. The outside expectation is with respect to the probability distribution $Q(k)$, and the inside expectation is with respect to the transition probabilities of the Markov chain.

Below we present a bound for R_* for a generic network.

Theorem 2 *Consider a network with degree connectivity distribution $P(k)$. Assume $c_0 > c_1$. Then R_* defined in Definition 5 has the following upper bound,*

$$R_* \leq \frac{\beta}{\delta} \sum_{k=1}^{\min(\lfloor c_0/c_2 \rfloor, n)} \frac{k^2 P(k)}{\sum_{k=1}^{n} kP(k)} \tag{S48}$$

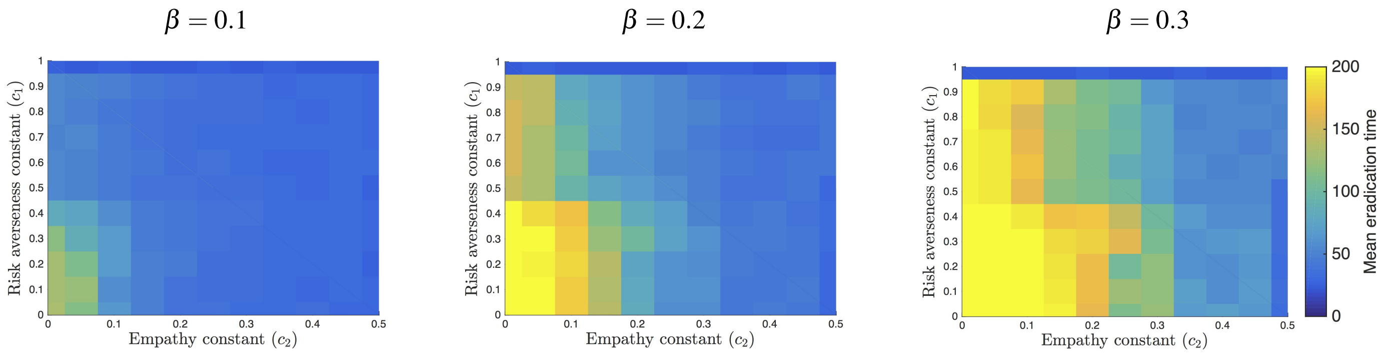

Figure S3. Effect of risk averseness c_1 and empathy c_2 constants on mean eradication time. We fix the healing rate to $\delta = 0.2$ and the population size to $n = 100$. The infection rate β values equal to 0.1,0.2, and 0.3 for figures left, middle, and right, respectively. We let $c_0 = 1$ for each figure. The axes in the figures correspond to the constant values of c_1 and c_2. For a given value of c_1 and c_2, we generate 50 scale-free networks using the preferential attachment algorithm and run the stochastic disease network game for 200 steps for each network. Each run starts with all individuals infected. The grid color represents the average eradication time among runs where we let eradication time be equal to 200 if the disease is not eradicated. The eradication time decreases as c_1 or c_2 increases in the region where disease is eradicated fast, i.e., $R_* > 1$.

Proof: *First note that the only difference between the R_0 definition and R_* is the difference in probability distributions of selecting the initial infected individual. Hence, the bound in (S43) applies to R_* which we repeat here for convenience,*

$$R_* \leq \frac{\beta}{\delta} E\left[|\mathcal{N}_i| \mathbf{1}(c_0 > c_2 |\mathcal{N}_i|)\right] \tag{S49}$$

where now the expectation is with respect to the distribution $Q(k)$. Therefore, the expectation on the right hand side is equal to the following,

$$R_* \leq \frac{\beta}{\delta} \sum_{k=1}^{n} k \mathbf{1}(k < \frac{c_0}{c_2}) Q(k) \tag{S50}$$

Bound in (S48) follows by using the definition of $Q(k) = \frac{kP(k)}{\sum_k kP(k)}$.

F R_* bound scale-free

Corollary 2 *Consider a random scale free network where the degree distribution follows $P(k) \sim k^{-\gamma}$ for $\gamma = 2$. Then R_* given by Definition 5 has the following upper bound,*

$$R_* \leq \frac{\beta}{\delta} \frac{\min(\lfloor c_0/c_2 \rfloor, n)}{\log(n)}. \tag{S51}$$

Proof: *We directly use the bound in (S48) and substitute in $P(k) = L(-2,n)k^{-2}$ where $L(-2,n) = (\sum_{k=1}^{n} k^{-2})^{-1}$ is the normalization constant for the scale-free distribution*

$$R_* \leq \frac{\beta}{\delta} \sum_{k=1}^{\min(\lfloor c_0/c_2 \rfloor, n)} \frac{L(-2,n)}{L(-2,n)\sum_{l=1}^{n} l^{-1}} = \frac{\beta}{\delta} \sum_{k=1}^{\min(\lfloor c_0/c_2 \rfloor, n)} \frac{1}{\sum_{l=1}^{n} l^{-1}}. \tag{S52}$$

The bound in (S51) follows by noting that $\sum_{l=1}^{n} l^{-1} \approx \log(n)$.

G Mean eradication time

See Figure S3 for the effect of parameters c_1, c_2, β on eradication time.

H Average infectivity levels

See Figure S4 for the effect of parameters c_1, c_2, β on infectivity level.

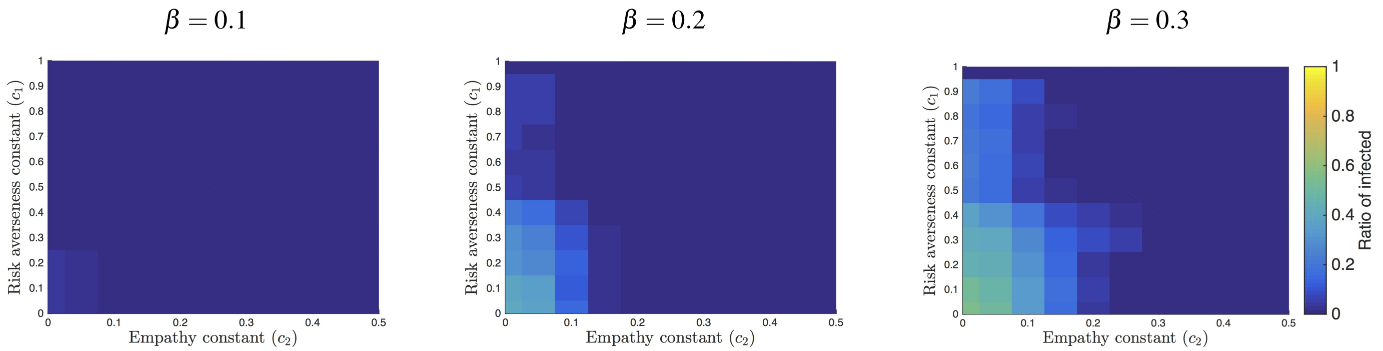

Figure S4. Effect of risk averseness c_1 and empathy c_2 constants on average infectivity level. We fix the healing rate to $\delta = 0.2$ and the population size to $n = 100$. The infection rate β values equal to 0.1,0.2, and 0.3 for figures left, middle, and right, respectively. We let $c_0 = 1$ for each figure. The axes in the figures correspond to the constant values of c_1 and c_2. For a given value of c_1 and c_2, we generate 50 scale-free networks using the preferential attachment algorithm and run the stochastic disease network game for 200 steps for each network. Each run starts with all individuals infected. The grid color represents the average of infectivity level at time $t = 200$ among runs. Increasing c_1 reduces the average number of infected in the population whether the disease is endemic or not.

I Critical empathy thresholds for modified scale-free networks

In the preferential attachment model, used in Figures 4-6 of the main text, a node is added at each step and is connected to an existing node selected randomly proportional to their degree–see the Barabasi-Albert algorithm[1]. A modification to this algorithm is when each added node is connected to two existing nodes selected randomly proportional to their degree. This modified algorithm generates scale-free networks with an expected scaling degree of $\gamma = 2.3$ while the non-modified preferential attachment algorithm generates an expected scaling degree of $\gamma = 2$.

In Figure S5, we test the accuracy of the bounds for R_0 and R_* in equations (S30) and (S48) (equations (1) and (4) in the main text) for scale-free networks generated according to the modified preferential attachment algorithm. Using these inequalities and substituting for $P(k) = L(-2.3, n)k^{-2.3}$ where $L(-2.3, n)$ is a normalizing constant, we solve for empathy constant values c_2 that make the right hand side of equations (1) and (4) less than one – see the caption of Fig. S5 for the critical empathy values. We note that the closed form critical thresholds of the empathy constant c_2 in equations (S45) and (S51) (equations (3) and (6) of main text) are obtained when the network is scale-free with scaling degree $\gamma = 2$. The critical empathy values obtained for $\gamma = 2.3$ are greater than the critical empathy values when $\gamma = 2$. Numerical experiments show that the critical empathy constants that make $R_* < 1$ are still good indicators of fast disease eradication.

J Sensitivity to initial level of infectivity

We assess the robustness of the critical empathy threshold to initial conditions by considering initial infectivity levels of $\{5\%, 20\%, 50\%\}$ in Figure S6. Besides the difference in initial conditions, the numerical setup is identical to Figure 6 in main text. The numerical experiments confirm that the critical empathy threshold for R_* in (S51) is a good indicator for disease eradication. There are only a few runs which fail to eradicate the disease within 200 time steps when the critical empathy for $R_* < 1$ is exceeded – refer to the caption of Figure S6 for details.

K Epidemic threshold when $c_2 = 0$

If $c_2 = 0$, an MMPE action profile at time t can simply be written as follows,

$$a_i^*(t) = \mathbf{1}\left(c_0 > c_1(1 - s_i(t)) \sum_{j \in \mathcal{N}_i} s_j(t)\right). \tag{S53}$$

That is, the infected will socialize normally at all times and a susceptible agent will socialize if the number infected around is less than $\frac{c_0}{c_1}$. Given the MMPE profile above, the infection probability of a susceptible individual i is given by

$$p_{01}^i(t) = P(s_i(t+1) = 1 | s_i(t) = 0) = 1 - \prod_{j \in \mathcal{N}_i} (1 - \beta a_i^*(t)) s_j(t). \tag{S54}$$

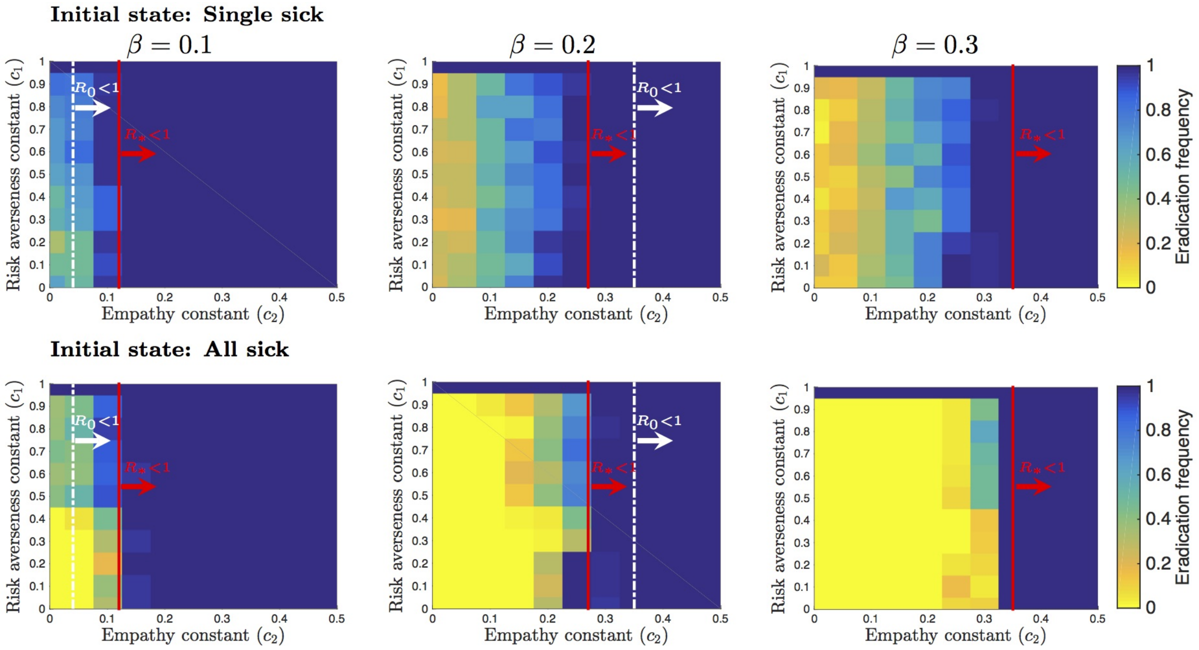

Figure S5. Effect of risk averseness c_1 and empathy c_2 constants on disease eradication with scale-free networks with higher scaling degree. We consider $n = 100$ individuals, and let $\delta = 0.2$, and $c_0 = 1$. The scale-free network is generated according to the modified preferential attachment algorithm resulting in a scale-free network with average scaling degree of $\gamma = 2.3$. The average node degree is 3.94. The critical values of c_2 $\{0.04, 0.35, 1.01\}$ that make $R_0 < 1$ for $\beta = \{0.1, 0.2, 0.3\}$ calculated using equation (S30) are marked with white dotted dashed lines. The critical values of the empathy constant, $c_2 = \{0.12, 0.27, 0.35\}$ (marked with red solid lines), that make $R_* < 1$ for $\beta = \{0.1, 0.2, 0.3\}$ calculated using equation (S48) are still accurate in determining fast eradication.

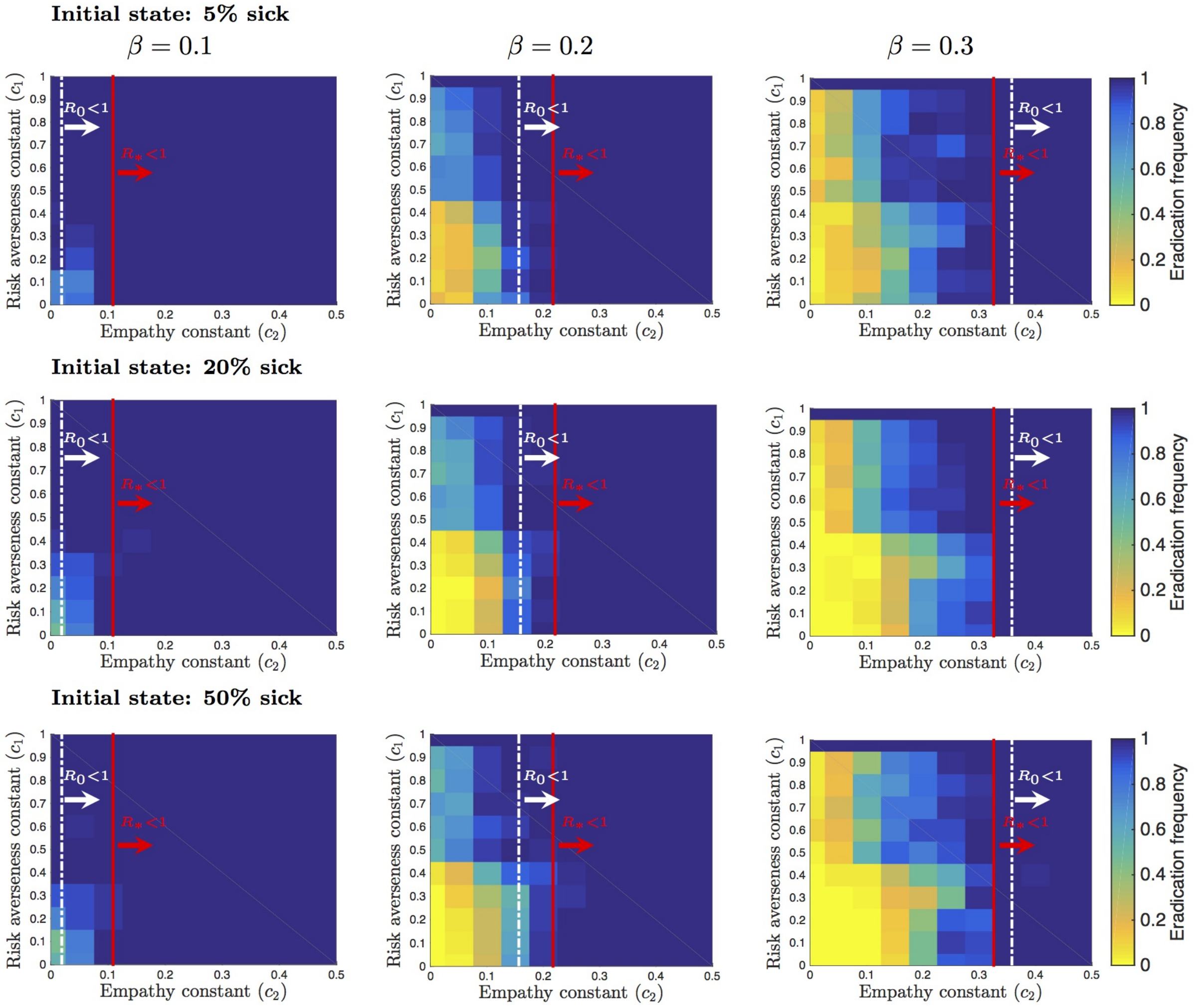

Figure S6. Effect of risk averseness c_1 and empathy c_2 constants on disease eradication when initial number of infected is 5% (top), 20% (middle), and 50% (bottom) of the population. We consider $n = 100$ individuals, and let $\delta = 0.2$, and $c_0 = 1$. For a given value of c_1 and c_2, we generate 50 scale-free networks using the preferential attachment algorithm and run the stochastic disease network game for 200 steps for each network. The grid color represents the ratio of runs in which disease is eradicated within 200 steps. For figures left, middle, and right the eradication of threshold $\beta\lambda_{max}(A)/\delta$ is equal to 2.65, 5.3, and 8, respectively. The critical values of c_2 $\{0.02, 0.16, 0.36\}$ that make $R_0 < 1$ for $\beta = \{0.1, 0.2, 0.3\}$ calculated using equation (S45) are marked with white dotted dashed lines. The critical values of the empathy constant, $c_2 = \{0.11, 0.22, 0.33\}$, that make $R_* < 1$ for $\beta = \{0.1, 0.2, 0.3\}$ calculated using equation (S51) are accurate in determining fast eradication for any value of c_1 (marked with red solid lines). The rate of disease eradication is above 95% among 50 trials when the value of the empathy constant is above the threshold that makes $R_* < 1$. That is, for c_2 that make $R_* < 1$ there are only a few trials that fail to eradicate the disease within 200 steps which are observed when $\beta = 0.1$ and 20% initially infected, and when $\beta = \{0.2, 0.3\}$.

We can bound the above conditional probability as follows by using the relation $1-\prod_{j\in\mathcal{N}_i}(1-\beta a_i a_j s_j(t)) \le \beta a_i \sum_{j\in\mathcal{N}_i} a_j s_j(t)$,

$$p^i_{01}(t) \le \beta a^*_i(t) \sum_{j\in\mathcal{N}_i} s_j(t). \tag{S55}$$

Next we obtain a n-state approximation of the Markov chain dynamics – see for a similar approximation[2]. Define the probability of infection of individual i at time t $p_i(t) = E[s_i(t)]$. Let $p(t) \in [0,1]^n$ be n infection probability vector of the population. By law of total probability,

$$p_i(t+1) = p_i(t)(1-\delta) + (1-p_i(t))p^i_{01}(t). \tag{S56}$$

Using the upper bound above for $p^i_{01}(t)$ and noting that $a^*_i = \mathbf{1}(c_0 > \beta c_1 \sum_{j\in\mathcal{N}_i} s_j(t))$ from (S53) if $s_i(t) = 0$, we obtain

$$p_i(t+1) \le p_i(t)(1-\delta) + (1-p_i(t))\beta\mathbf{1}\left(c_0 > c_1 \sum_{j\in\mathcal{N}_i} s_j(t)\right) \sum_{j\in\mathcal{N}_i} s_j(t). \tag{S57}$$

We now approximate the above bound by replacing $s_j(t)$ terms with $p_j(t)$ to get

$$p_i(t+1) \approx p_i(t)(1-\delta) + (1-p_i(t))\beta\mathbf{1}\left(c_0 > c_1 \sum_{j\in\mathcal{N}_i} p_j(t)\right) \sum_{j\in\mathcal{N}_i} p_j(t). \tag{S58}$$

When we linearize the dynamics of $p(t)$ defined by (S58) around the fixed point origin 0_n, i.e., the state of disease eradication $p_i(t) = 0$ for all i, we get the following,

$$p(t+1) = ((1-\delta)I_n + \beta A)p(t) \tag{S59}$$

where I_n is the $n\times n$ identity matrix and $A \in [0,1]^{n\times n}$ is the adjacency matrix of the contact network, i.e., its ijth element $A_{ij} = 1$ if $j \in \mathcal{N}_i$, otherwise $A_{ij} = 0$. The approximate disease dynamics in (S59) is linear. Furthermore, the origin 0_n is a globally stable fixed point if and only if $\lambda_{max}((1-\delta)I_n + \beta A) < 1$[3]. We can equivalently write this condition as $\frac{\beta}{\delta}\lambda_{max}(A) < 1$. This result implies that the state of disease eradication is unstable if $\frac{\beta}{\delta}\lambda_{max}(A) > 1$. Note that this threshold does not depend on the risk averseness constant c_1. This implies that risk aversion cannot by stop an outbreak when $c_2 = 0$, except in the extreme case of $c_0 < c_1$.

References

1. Barabási, A.-L. & Albert, R. Emergence of scaling in random networks. *Science* **286**, 509–512 (1999).
2. Paarporn, K., Eksin, C., Weitz, J. S. & Shamma, J. S. Epidemic spread over networks with agent awareness and social distancing. In *Proceedings of the 53rd Annual Allerton Conference on Communications, Control, and Computing* , 51–57 (Allerton, Illinois, USA, 2015).
3. Van Mieghem, P., Omic, J. & Kooij, R. Virus spread in networks. *IEEE/ACM Transactions on Networking* **17**, 1–14 (2009). DOI 10.1109/TNET.2008.925623.
